# High-Resolution Genetic Mapping Combined with Transcriptome Profiling Reveals That Both Target-Site Resistance and Increased Detoxification Confer Resistance to the Pyrethroid Bifenthrin in the Spider Mite *Tetranychus urticae*

**DOI:** 10.3390/biology11111630

**Published:** 2022-11-07

**Authors:** Berdien De Beer, Marilou Vandenhole, Christine Njiru, Pieter Spanoghe, Wannes Dermauw, Thomas Van Leeuwen

**Affiliations:** 1 Department of Plants and Crops, Faculty of Bioscience Engineering, Ghent University, Coupure Links 653, 9000 Ghent, Belgium; 2 Plant Sciences Unit, Flanders Research Institute for Agriculture, Fisheries and Food (ILVO), Burg. Van Gansberghelaan 96, 9820 Merelbeke, Belgium

**Keywords:** bifenthrin, pyrethroids, target-site resistance, metabolic resistance, *Tetranychus urticae*, carboxyl/choline esterases, UDP-glycosyl transferases

## Abstract

**Simple Summary:**

The two-spotted spider mite *Tetranychus urticae* is an important pest on agricultural crops worldwide. The widespread application of pyrethroid acaricides—such as bifenthrin—to control this pest has resulted in the development of resistance. Previous research has associated mutations in the voltage-gated sodium channel (VGSC), as well as increased detoxification, with bifenthrin resistance. Here, we performed a bulked segregant analysis to unbiasedly map resistance loci conferring resistance and found two genomic loci (QTL1 and QTL2) underlying bifenthrin resistance. The VGSC is located at QTL2, which harbors the resistance-conferring L1024V mutation. The presence of a second QTL suggested that a second resistance mechanism must be involved, and this was further investigated with a differential gene expression analysis. Multiple genes encoding detoxification enzymes, including carboxyl/choline esterases (CCEs), cytochrome P450 monooxygenases and UDP-glycosyl transferases (UGTs), were more highly expressed in bifenthrin-resistant strains. A selection of these enzymes (CCE58, CCEinc18, teturUGT10 and teturUGT29) were functionally expressed. CCEinc18 was shown to metabolize bifenthrin, while teturUGT10 could glycosylate bifenthrin-alcohol. In conclusion, our findings suggest that bifenthrin resistance in the spider mite *T. urticae* is mediated by both target-site and metabolic mechanisms that may act in synergy.

**Abstract:**

Pyrethroids are widely applied insecticides in agriculture, but their frequent use has provoked many cases of resistance, in which mutations in the voltage-gated sodium channel (VGSC), the pyrethroid target-site, were shown to play a major role. However, for the spider mite *Tetranychus urticae*, it has also been shown that increased detoxification contributes to resistance against the pyrethroid bifenthrin. Here, we performed QTL-mapping to identify the genomic loci underlying bifenthrin resistance in *T. urticae*. Two loci on chromosome 1 were identified, with the VGSC gene being located near the second QTL and harboring the well-known L1024V mutation. In addition, the presence of an L925M mutation in the VGSC of a highly bifenthrin-resistant strain and its loss in its derived, susceptible, inbred line indicated the importance of target-site mutations in bifenthrin resistance. Further, RNAseq experiments revealed that genes encoding detoxification enzymes, including carboxyl/choline esterases (CCEs), cytochrome P450 monooxygenases and UDP-glycosyl transferases (UGTs), were overexpressed in resistant strains. Toxicity bioassays with bifenthrin (ester pyrethroid) and etofenprox (non-ester pyrethroid) also indicated a possible role for CCEs in bifenthrin resistance. A selection of CCEs and UGTs were therefore functionally expressed, and CCEinc18 was shown to metabolize bifenthrin, while teturUGT10 could glycosylate bifenthrin-alcohol. To conclude, our findings suggest that both target-site and metabolic mechanisms underlie bifenthrin resistance in *T. urticae*, and these might synergize high levels of resistance.

## 1. Introduction

**Figure 1:**
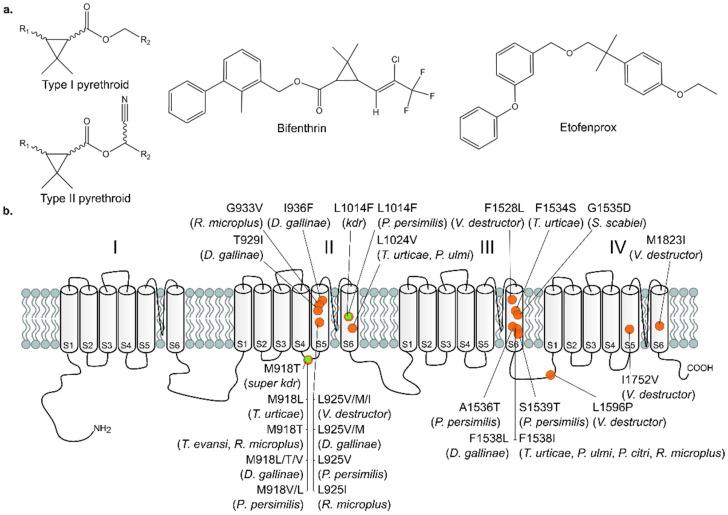
Pyrethroids and their target-site, the voltage gated sodium channel (VGSC; drafted from Van Leeuwen et al. [14]). (**a**) Chemical structure of Type I and II pyrethroids, the ester-pyrethroid bifenthrin and the non-ester pyrethroid etofenprox. (**b**) Schematic presentation of the VGSC, composed of four repeat domains (I–IV), each containing six membrane spanning helices (S1–S6). The positions of resistance mutations—found in *T. urticae* and other Acari species—involved in pyrethroid resistance are depicted with orange circles, while the *kdr* and *super kdr* mutations, identified in many insects, are depicted with green squares (numbering according to *Musca domestica*) [14,15,16,17,18,19,20,21,22,23,24,25,26,27,28,29,30,31,32].

Pyrethroids are one of the most frequently used insecticides in the world. They are highly selective with a limited mammalian toxicity and have a low environmental persistence [1,2,3,4,5,6]. These synthetic pesticides are related to the natural pyrethrins present in *Chrysanthemum cinerariaefolium* (pyrethrum daisy) and belong to the IRAC Mode of Action group 3A ‘Pyrethroids and Pyrethrins’ [1,5,7]. This group is further divided into subcategories: Type I and Type II pyrethroids—ester pyrethroids without or with an α-cyanogroup, respectively—and non-ester pyrethroids. These pyrethroid subcategories show distinct biological responses (reviewed in Khambay and Jewess [1] and Soderlund [8]), although, similar to DDT and its analogs, they all modulate the voltage-gated sodium channels (VGSCs) (Figure 1) [6]. These channels form a pore in the membrane that is highly selective for sodium ions, and as the name suggests, the opening and closing of the VGSCs are voltage-dependent [9,10]. Upon membrane depolarization, the VGSC gets activated and opens, which results in further depolarization of the membrane. The effects of Type I and Type II pyrethroids depend on the preference to binding closed or opened VGSCs. Type I pyrethroids, such as the acaricide bifenthrin ((2-methyl-3-phenylphenyl)methyl 3-[(Z)-2-chloro-3,3,3-trifluoroprop-1-enyl]-2,2-dimethylcyclopropane-1-carboxylate), cause hyperactivity, repetitive neuronal discharges and membrane depolarization and bind preferentially to closed VGSCs [1]. The non-ester pyrethroid etofenprox (1-((2-(4-ethoxyphenyl)-2-methylpropoxy)methyl)-3-phenoxybenzene) has the same biological effect as Type I pyrethroids [11,12], although the literature suggests that the binding site of etofenprox is similar to those of Type II pyrethroids [13]. Type II pyrethroids, like cypermethrin, prefer binding to open VGSC and are characterized by nerve depolarization followed by paralysis [1].

Pyrethroids are mainly used in agriculture for controlling insect and mite pests [6,33] but are also frequently applied in vector-borne disease control and veterinary medicine [34,35,36]. Due to their widespread application and long-term usage, many cases of pyrethroid resistance have been reported, including for the two-spotted spider mite *Tetranychus urticae* (Chelicerata: Acari) [37,38]. This species is highly polyphagous and an important pest on agricultural crops worldwide [3,39]. Due to its high fecundity, arrhenotokous reproduction and short life cycle, it rapidly develops resistance against acaricides [3,40]. Resistance mechanisms can be classified into toxicokinetic mechanisms—including decreased exposure to the toxic substance due to increased metabolism, decreased penetration, sequestration or increased excretion—and toxicodynamic mechanisms such as decreased sensitivity of the target-site towards the insecticide [31]. In the case of pyrethroid resistance, toxicodynamic resistance has been shown to be the most important mechanism [1], and “knockdown resistance” or *kdr* was first described in houseflies (*Musca domestica*) by Busvine [41]. The *kdr* trait causes a loss of sensitivity to DDT and pyrethroids due to a reduction in the binding of pyrethroids to the VGSC. In several cases, the *kdr* trait is accompanied by a second resistance trait named *super-kdr*, which confers an even higher resistance to pyrethroids [42]. In follow-up studies, the reduced insecticide binding was attributed to the presence of mutations in the VGSC, with the *kdr* mutation (L1014F) being documented globally in many major arthropod pests and disease vectors (reviewed in Rinkevich, Du and Dong [10]). For the *Tetranychus* species, several mutations in the VGSC have also been associated with decreased sensitivity towards Type I and Type II pyrethroids: point mutations M918L/T or L925M in the intracellular linker connecting transmembrane segments IIS4 and IIS5 (where M918L was found in combination with F1534S in IIIS6), L1024V at the terminus of the IIS6 transmembrane segment of the sodium channel and F1538I in domain IIIS6 (*M. domestica* numbering) [16,17,18,19,20,21] (Figure 1) (Table S1). Similar VGSC mutations have also been reported for other spider mites such as *Panonychus citri* and *Panonychus ulmi*, but also in other mites and ticks, including the cattle tick *Rhipicephalus microplus*, the *Varroa* mite, the poultry red mite *Dermanyssus gallinae* and predatory mites such as *Phytoseiulus persimilis* [22,25,26,27,28,29,30,31].

Although target-site resistance plays a major role in the pyrethroid resistance of *T. urticae*, Riga et al. showed that the L1024V and F1538I+A1215D mutations in the VGSC only account for a part of the bifenthrin resistance phenotype [19], suggesting that toxicokinetic mechanisms may also be involved in pyrethroid resistance. In both a Turkish and a Belgian *T. urticae* strain, bifenthrin resistance was correlated with increased carboxyl/choline esterase (CCE) activity, and an increased bifenthrin metabolism was documented in the Belgian strain [43,44,45]. Furthermore, synergism tests in a bifenthrin-resistant *T. urticae* strain with the esterase inhibitor S,S,S-tributyl-phosphorotrithioate (DEF) showed an increase in bifenthrin toxicity [45,46,47]. By evaluating the general esterase activity and performing synergism tests with triphenyl phosphate, Yang et al. found similar results for the detoxification of the pyrethroids bifenthrin and λ-cyhalothrin [48,49]. Although the majority of *T. urticae* pyrethroid resistance reports implicate a role for CCEs in resistance [44,45,47,49,50], increased cytochrome P450 monooxygenase (CYP) and glutathione S-transferase (GST) activities have also been associated with spider mite resistance against pyrethroids [17,44,48].

In this study, we aimed to elucidate the resistance mechanisms of *T. urticae* against bifenthrin. Toxicity tests with an ester-pyrethroid bifenthrin and a non-ester pyrethroid etofenprox, together with the presence of target-site mutations in the VGSC, revealed the complexity of bifenthrin resistance. We therefore performed a bulked segregant analysis (BSA) as an unbiased approach to identify the genomic regions underlying resistance. The VGSC gene was located in one of the identified regions (QTL2), while a second region (QTL1) suggested that an additional resistance mechanism was at play. These additional players were further investigated by performing differential gene expression analysis between several bifenthrin-resistant and -susceptible spider mite strains.

## 2. Materials & Methods

### 2.1. Acaricides

The acaricides utilized in this study were commercial formulations of bifenthrin (Talstar; 80 g L^−1^ SC) and etofenprox (Therbonal; 28.75 g L^−1^ EC).

### 2.2. Mite Strains and Husbandry

The *T. urticae* strains used in this study were MR-VL, El Juan, SR6, ES1, IT3, ROS-IT, UK4 and RO1. Two color morphs have been described for *T. urticae* [51] and are referred to here as ‘green’ and ‘red’. The red *T. urticae* strain MR-VL was originally collected in a greenhouse at Ghent University and already showed high resistance to bifenthrin, dicofol and fenbutatin oxide (FBO) in previous studies [44,52]. During this study, MR-VL was maintained on a selection pressure of 400 mg L^−1^ bifenthrin and sprayed on bean plants with a hand-held spraying device (Birchmeier, Stetten, Switzerland) until runoff to avoid contamination. The red strains El Juan and SR6 were collected on tomato plants in greenhouses in Malaga (Spain) and in Italy, respectively [53]. ES1 and IT3 are both red spider mite strains, collected on strawberry in Spain and citrus in Italy, respectively [54]. The green *T. urticae* strains ROS-IT (synonymous with the IT2 used in Xue et al. [54]), UK4 and RO1 were all collected in 2017 [54]. ROS-IT and RO1 were both collected on rose, in Italy and Romania, respectively, while UK4 was collected in the United Kingdom on strawberry.

This study also uses inbred (suffix i) strains (MR-VLi, SR6i and ROS-ITi) derived from subpopulations of the MR-VL, SR6 and ROS-IT cultures, which were made inbred by seven to eight generations of consecutive mother–son mating, as described by Bryon et al. [55]. All inbred strains used in this study were described earlier in Kurlovs et al. [20] and in De Beer et al. [52].

All mite strains were maintained on potted kidney bean plants (*Phaseolus vulgaris* L. cv. Prelude) in a climatically controlled room or incubator at 25 (±0.5) °C and 60% relative humidity and under a 16:8 h light:dark photoperiod. During BSA experiments, the experimental mite populations derived from a SR6i × MR-VLi cross were maintained on potted kidney bean plants placed in mite-proof cages (BugDorm-4F4590DH, MegaView Science Co., Taichung, Taiwan) in a greenhouse at 25 °C and 60% relative humidity.

### 2.3. Toxicity Bioassays

The toxicity of bifenthrin and etofenprox was assessed in adulticidal bioassays, performed as previously described by Van Pottelberge et al. [56]. To summarize, 20–30 adult female mites were placed on 9 cm^2^ square bean leaf disks placed on wet cotton. Acaricide formulations were diluted with demineralized water, and at least five concentrations of the respective acaricide, and water as the control, were tested in four replicates. Using a Cornelis spray tower, 800 µL fluid was sprayed on the mites on each leaf-disk at a pressure of 1 bar (1.5 mg fluid deposition per cm^2^). Mortality was assessed after 48 h by scoring mites as dead when they could not walk normally after prodding with a camel’s hairbrush. Corresponding concentration-response curves, lethal concentrations for 50% of the subject (LC_50_) values, resistance ratios (RR) and 95% confidence intervals (CI) were calculated by probit analysis (PoloPlus, version 2.0, LeOra Software, Berkeley, CA, USA).

### 2.4. Screening for Resistance Mutations in the VGSC

Based on the gene expression data and DNA sequences available from this study (see Section 2.5 and Section 2.6 for sequencing and mapping details) and previous studies [52,54], all *T. urticae* strains used in this study were screened for the presence of VGSC mutations (M918L, L925M, L1024V, F1534S, F1538I; *M. domestica* numbering) previously shown to be associated with pyrethroid resistance in *T. urticae* [16,19,20]. Briefly, since the number of RNAseq read counts aligning with the VGSC gene was low for the strains El Juan, MR-VL, ES1, IT3, ROS-IT, UK4 and RO1, the presence of the aforementioned mutations was visually examined using the Integrative Genomics Viewer [57], and the mutation was considered present when the mutation was observed in at least 20% of the reads. In the case of ROS-ITi, SR6i and MR-VLi, genomic DNA sequences were available (from a previous study [52] and the current study), and the presence of VGSC mutations was assessed using the gVCF files used for BSA analyses.

### 2.5. Bulked Segregant Analysis

#### 2.5.1. Set-Up Bulk Segregant Analysis

An initial cross was made by placing 40 virgin females (teleiochrysalid stage) of the susceptible strain SR6i along with 18 adult males of the bifenthrin-resistant strain MR-VLi on a detached bean leaf. The resulting population expanded on detached bean leaves for three generations, until they were placed on potted bean plants in mite-proof cages (BugDorm-4F4590DH, MegaView Science Co., Taichung, Taiwan) and allowed to grow in a greenhouse at 25 °C and 60% relative humidity. After the fourth generation of expansion on the bean plants, a toxicity bioassay with bifenthrin was performed on the segregating population, and immediately afterwards (11 weeks since the initial cross), the population was split into 10 subpopulations of 500 female adult mites each. After two generations of population increases, each subpopulation was split into a control line, allowed to grow without selection pressure, and a paired selected line, selected with increasing concentrations of bifenthrin by placing 500 females on sprayed plants. This set-up resulted in 10 paired populations. Selection with bifenthrin was carried out by spraying uninfested bean plants with a hand-held spraying device (Birchmeier, Switzerland) until runoff—starting with a concentration of 38 mg L^−1^ bifenthrin—and transferring mites to freshly sprayed plants. The concentration of bifenthrin was gradually raised to 400 mg L^−1^. The effectiveness of acaricide selection was assessed prior to DNA extraction by testing each subpopulation, control and selected, with a discriminating dose of 2500 mg L^−1^ bifenthrin. Differences in the survival rate between the selected and control replicates were analyzed in R (version 4.0.4) using a linear mixed model (fixed factor = ‘treatment’, random factor = ‘replicate’) and a Satterthwaite’s approximation to calculate adjusted *p*-values.

Finally, 800 mites were collected per population (12th to 14th generation on plants with or without selection pressure), and genomic DNA (gDNA) was collected by a chloroform-phenol extraction, as previously described [58,59], and purified using the E.Z.N.A.^®^ Cycle Pure Kit DNA purification kit (OMEGA Bio-tek, Norcross, GA, USA). The quality and quantity of the gDNA samples were assessed using a Denovix DS-11 spectrophotometer (DeNovix, Willmington, DE, USA) and by running a 2% agarose gel electrophoresis (30 min at 100 V). The genomic DNA of the parental strains MR-VLi and SR6i was extracted using the same method, but prior to the BSA set-up.

#### 2.5.2. DNA Sequencing and Bioinformatic Analyses

Library construction (using the Truseq Nano DNA sample Preparation Kit (Illumina, San Diego, CA, USA)) and sequencing were performed at the Huntsman Cancer Institute of the University of Utah (Salt Lake City, Utah, USA) for the experimental populations and the parental strains. The samples of the experimental BSA populations were sequenced on a NovaSeq 6000, generating paired reads with lengths of 150 bp and mean insert sizes of 450 bp. A HiSeq 2500 instrument was used for sequencing the parental strains MR-VLi and SR6i to generate paired reads with lengths of 125 bp and mean insert sizes of 450 bp. The genomic sequence reads for all populations (bifenthrin-selected and unselected control) have been deposited in the NCBI Sequence Read Archive under BioProject (PRJNA895330), while the genomic sequence reads for the parental strains (MR-VLi and SR6i) were from prior public releases to the NCBI Sequence Read Archive (PRJNA779219 for MR-VLi and SR6i).

For each sample, the reads were aligned to the three-pseudochromosome assembly of *T. urticae* [60,61] using the default settings of the ‘Burrows–Wheeler Aligner’ (BWA) (version 0.7.12-r1039) and processed into position-sorted BAM files using ‘SAMtools’ (version 1.9.1) [62]. Following the recommendations described in the ‘Genome Analysis Toolkit’ (GATK) best practices pipeline [63], duplicates were marked using ‘Picard tools’ (version 2.20.4-SNAPSHOT; https://broadinstitute.github.io/picard, accessed on 8 March 2022), followed by indel realignment (‘LeftAlignIndels’) with GATK (version 4.1.4.1) [64]. Joint variant calling across all 20 populations and the parental strains and subsequent BSA analysis were carried out as described earlier in De Beer et al. [52]. Using the final gVCF file as the input, *T. urticae* genes located within 100 kb of the QTL2 region were visualized using ‘Gviz’ (version 1.40.1) [65] and the gff3 annotation of the *T. urticae* London genome [60,61]. A principal component analysis (PCA) was performed with the R-package ‘prcomp’ (version 2.3.0), as described in Snoeck et al. [66]. Briefly, a correlation matrix containing individual SNP frequencies for each population was used as the input for ‘prcomp’. Only SNPs that differentiated the two parental lines and that were present in all treatments (BIF-selected (BIF) and control (CON)) were included in this correlation matrix. A two-dimensional PCA plot was created using the function ‘autoplot’ in the R package ‘ggplot2’ (version 2.1.0) [67].

#### 2.5.3. Potential Effect of Variant Alleles in Coding Sequences

To predict the potential effect of coding sequence changes in the QTL regions identified in the BSA assays, the coding effects of SNPs and small indels (with “HIGH” or “MODERATE” impact) found in the GATK analysis were predicted using ‘SnpEff’ (version 5.0c) [68], with a *T. urticae* coding sequence database derived from the 23 June 2016 annotation (available from the Online Resource for Community Annotation of Eukaryotes—ORCAE [69]) and a *T. urticae* ‘SnpEff’ database built using the three-pseudochromosome assembly reference genome [61]. Within the ‘SnpEff’ package, the SNPsift toolbox [70] was used to filter the ‘SNPeff’ output for variants present in the resistant parent line, absent in the susceptible parent line and enriched in all selected populations (i.e., the allelic depth of the variant allele being higher than the allelic depth of the reference allele).

### 2.6. Differential Gene Expression

#### 2.6.1. RNA Extraction and Sequencing

In order to gain more insight in the detoxification mechanisms possibly involved in bifenthrin resistance, a differential gene expression analysis was performed on a collection of strains for which gene expression data were available (MR-VL, El Juan, ES1, ROS-IT, UK4, IT3 and RO1). Briefly, two weeks prior to RNA extractions, the strains were transferred to unsprayed fresh bean plants. RNA was extracted in four independent replicates from 80–120 female adult mites according to the RNeasy Mini Plus Kit—Quick Start Protocol (Qiagen, Belgium). After elution in 30 µL RNA-free water, the concentrations and purity of the RNA samples were measured using a DS-11 spectrophotometer (DeNovix, Willmington, DE, USA). RNA integrity was checked via gel electrophoresis (1% agarose gel—30 min at 100 V). All RNA samples were stored at −80 °C prior to sequencing.

Library construction and sequencing were performed at the High-Throughput Genomics and Bioinformatic Analysis Shared Resource of the Huntsman Cancer Institute (University of Utah, Salt Lake City, UT, USA). Illumina libraries, constructed with the TruSeq Stranded mRNA Library Preparation Kit with polyA selection (Illumina, San Diego, California, USA), were sequenced via Illumina’s HiSeq2500 sequencing technology to generate strand-specific, paired-end reads of 2 × 125 bp. The RNA reads for MR-VL, El Juan and UK4 have been deposited in the NCBI Sequence Read Archive under BioProject (PRJNA896577), while the RNA reads for ES1, IT3, ROS-IT (synonymous with the IT2 used in Xue et al. [54]) and RO1 are available in the Gene Expression Omnibus Repository, with accession number GSE146593.

#### 2.6.2. RNA Read Mapping and Principal Component Analysis

The quality of the RNAseq reads was verified using ‘FastQC’ (version 0.11.9) [71]. The RNA reads of the samples were aligned to the *T. urticae* three-chromosome genome assembly [61] using the two-pass alignment mode of ‘STAR’ (version 2.7.9a), with a maximum intron size set to 20 kb [72]. The resulting BAM files were subsequently sorted by chromosomal coordinates and indexed using ‘SAMtools’ (version 1.15) [73]. ‘HTSeq’ (version 0.11.2) was used to perform read-counting on a per-gene basis with the default settings and “—stranded yes and—feature exon” [74]. In order to assess gene expression variation within and between groups, a principal component analysis (PCA) was performed using the count files as the input for the R-package ‘DESeq2’ (version 1.34.0) [75]. Briefly, the read counts were normalized via the regularized-logarithm (rlog) transformation function of ‘DESeq2’, the PCA was calculated and plotted for the 5000 most variable genes across all RNA samples using the ‘DESeq2’ function PlotPCA.

#### 2.6.3. Differential Expression (DE) Analysis

Differential expression (DE) analysis was performed using ‘DESeq2’ (version 1.34.0) [75], based on the total per-gene read counts generated by ‘HTSeq’ (see Section 2.6.2). First, differentially expressed genes (DEGs), fold change (FC) ≥2 and a Benjamini–Hochberg adjusted *p*-value (*p* adj < 0.05) were determined pairwise between bifenthrin-resistant and -susceptible mite populations: red strain comparisons: ES1 vs. IT3, MR-VL vs. IT3, ES1 vs. El Juan and MR-VL vs. El Juan and green strain comparisons: ROS-IT vs. RO1 and ROS-IT vs. UK4. Those genes that were differentially expressed in all pairwise comparisons were considered as the core set of differentially expressed genes related to bifenthrin resistance (although, strictly taken, this is not an absolute requirement for a gene to be involved in bifenthrin resistance). A second approach consisted of determining the differentially expressed genes between all resistant strains and all susceptible strain (ES1, MR-VL and ROS-IT vs. IT3, El Juan, RO1 and UK4) using ‘DESeq2’.

Gene expression heatmaps of the six pairwise differential expression comparisons were built using the resulting log_2_FC and the R-packages ‘limma’ (version 3.46.0) and ‘gplots’ (version 3.1.1), as described previously [54]. Venn diagrams were created according to Bardou et al. [76], and a volcanoplot was created using the ‘ggplot2’ package [67].

### 2.7. De Novo Transcriptome Assembly of MR-VL and the Mining of Transcripts Encoding CCEs

The differential gene expression analysis (in Section 2.6) indicated that several CCE genes and fragments were overexpressed in bifenthrin-resistant strains. To identify the alleles in the bifenthrin-resistant strain MR-VL that correspond to these overexpressed CCE genes and fragments (according to the annotation of the London reference genome used for read mapping), we assembled a de novo transcriptome for the MR-VL strain using the CLC Genomics Workbench (version 11.0.1) with default settings and performed a BLASTn search against this transcriptome using the overexpressed CCE genes and fragments (from the London reference genome [60]) as the query. Those MR-VL alleles that had the highest identity with the overexpressed CCE genes/fragments were selected for recombinant expression, and the MR-VL full length alleles of *CCE58* and *CCEinc18* were derived using both the transcript and previously released genomic data of MR-VL (SRA accession SRX7472637).

### 2.8. Activity of CCE58 and CCEinc18

#### 2.8.1. Recombinant Expression of CCE58 and CCEinc18 in the *Pichia pastoris* System

The full-length MR-VL alleles of the carboxyl/choline esterases *CCE58* (MRVL_contig_6457) and *CCEinc18* (MR-VL_contig_1813) were extracted from the MR-VL transcriptome assembly (File S1) and recombinantly expressed using the eukaryotic *Pichia pastoris* expression system, as described in Wei et al. [77]. Briefly, signal peptide sequences were removed, and both CCE genes were codon-optimized for expression in yeast (*Pichia pastoris*) by GenScript (USA) (File S2). The CCE genes were subcloned via double digest with EcoRI and XbaI in the methanol-inducible yeast expression vector pPICZαA, which contains a secretion signal. Electrocompetent X-33 *P. pastoris* cells were transformed with 0.1 μg of linearized recombinant vector using a MicroPulser (Bio-Rad, Belgium) electroporator with the conditions set at the Pic program (2 kV) in a 0.2 cm electroporation cuvette (Bio-Rad, Belgium). After electroporation, the transformed cells were allowed to recover in 1 mL of sorbitol (1 M) for 1 h at 30 °C before plating out on selective YPD plates (for the composition of YPD plates, see Table S2) containing 0.1% Zeocin (Thermo Fisher Scientific, Waltham, MA, USA). Subsequently, in order to produce sufficient CCE58 and CCEinc18 proteins, selected Zeocin-resistant *P. pastoris* X-33 colonies containing the *CCE58*-pPICZαA and *CCEinc18*-pPICZαA constructs were inoculated in a 1 L BMGY medium (for the composition of the BMGY medium, see Table S2) and grown at 30 °C while shaking vigorously. Then, 48 h later, cells were harvested and resuspended in 1 L BMMY induction medium (for the composition of the BMMY medium, see Table S2) to start protein production. During a period of 48 h, the medium was spiked every 12 h with 1% methanol in order to maintain protein production. Finally, the medium containing proteins was collected via centrifugation, after which the proteins were precipitated overnight with 80% ammonium sulfate at 8 °C. The precipitated proteins were dissolved in 0.1 M PBS (pH 7.4), and salt was depleted via multiple buffer exchange steps using a Pierce™ Protein Concentrator (30 kDa, 5–20 mL, Thermo Fisher Scientific, USA). Desalted CCE proteins containing a 6X HIS-tag were purified via passage through a 10 mL Ni-NTA column (Qiagen, Hilden, Germany) and then washed and eluted with 0.1 M PBS (pH 7.4) containing serial dilutions of imidazole (from 25 to 250 mM). The final purified protein was analyzed by sodium dodecyl sulfate–polyacrylamide gel electrophoresis (SDS–PAGE) and Western blotting and stored as a 50% (*v*/*v*) glycerol solution.

#### 2.8.2. Kinetic Analysis of CCE58 and CCEinc18 with Model Substrates

Concentrations of recombinantly expressed CCE58 and CCEinc18 were determined using the Bradford method [78]. Esterase activities against two model substrates, 4-nitrophenyl acetate (4-NPA) and 1-naphthyl acetate (1-NA), were determined using spectrophotometric measurements in a 96-well microplate by an Eon microplate reader (Agilent, Santa Clara, CA, USA), as previously described [44]. Briefly, the reaction mixture to measure esterase activity towards 4-NPA consisted of 5 μL (1 μg) of CCE enzyme source, 175 μL of phosphate buffer (0.1 M, pH 7.5) and 20 μL of substrate (concentration in the well ranging from 50 to 1600 μm) in 10% (*v*/*v*) acetone, resulting in 200  μL of total reaction volume in the well. The rate of 4-nitrophenol formation was measured using an Eon microplate spectrophotometer (BioTek, France) at 405 nm and 30 °C, using its kinetic mode for 15 s during a 3 min incubation period. Reaction rates were corrected for spontaneous 4-nitrophenol formation of a control without an enzyme and converted into specific activity via a standard curve of 4-nitrophenol in sodium phosphate buffer (0.1 M, pH 7.5). For 1-NA, on the other hand, esterase activity was determined in a reaction mixture of 5 μL (1 μg) of enzyme source, 155 μL of phosphate buffer (0.1 M, pH 7.5) and 20 μL of freshly prepared filtered fast blue RR solution (1.5 g L^−1^). The reaction was initiated by adding 20 µL of 1-NA in 10% (*v*/*v*) acetone, resulting in 200 μL of total reaction volume in the well. The formation of the 1-naphthol–fast blue RR dye complex was measured as described above at 500 nm. Reaction rates were corrected for spontaneous 1-naphthol formation of a control without an enzyme and converted into specific activity via a standard curve of naphthol in sodium phosphate buffer (0.1 M, pH 7.5) with Fast Blue RR dye (1.5 g L^−1^). The kinetic parameters V_max_ and K_m_ were estimated from a Michaelis–Menten curve using the software SigmaPlot (version 14.5, Systat Software, San Jose, CA, USA).

#### 2.8.3. Bifenthrin Metabolism

The ability of the recombinant CCE58 and CCEinc18 proteins to metabolize bifenthrin was tested in vitro. The reaction was started by combining 10 µg of protein in 990 µL of sodium phosphate buffer (0.1 M, pH 7.5) and 10 µL of a 1000 mg L^−1^ bifenthrin stock solution in acetone, resulting in an equal concentration of 10 mg L^−1^ protein and bifenthrin in the final reaction mix. As a control, the same reaction mixture was used, except for the enzyme source, which was boiled for 10 min to ensure inactivation. This reaction mixture was incubated for 2 h at 30 °C in a shaking warm water bath. For CCEinc18, the amount of bifenthrin breakdown (substrate depletion) was calculated at 1, 2, 4 and 16 h of incubation. Following the incubation, bifenthrin was extracted with 5 mL of hexane and dried from excess buffer with a 0.5 cm sodium-phosphate drying column. The bifenthrin extract was brought back to a final volume of 5 mL using hexane. The bifenthrin concentration in the extracts was analyzed using an Agilent Technologies 6890 N gas chromatograph equipped with an Agilent Technologies 7683 Series autosampler injector, coupled to an electron capture detector (GC-ECD). Separation was performed on an HP-5MS (5% phenyl methyl siloxane) capillary column (30 m × 0.25 mm, 0.25 μm film thickness). The temperatures of the injector and detector were maintained at 200 °C and 250 °C, respectively. Helium was used as a carrier gas at a flow rate of 1.1 mL min^−1^, and the injections were made in the split mode with a split ratio of 52.7:1. The peak areas [Hz*s] of bifenthrin, detected at a retention time of 17.24 min, were used to quantify bifenthrin using a calibration curve (0.1–4 ppm). All experiments had at least four replicates. Bifenthrin depletion rates were calculated as nmol bifenthrin depletion per minute and per mg protein. Bifenthrin depletion is the difference between the means in nmol bifenthrin after an incubation of 120 min with 10 µg of the respective (in)active protein treatment, as compared to 120 min of incubation without protein supplementation. Differences in bifenthrin depletion rates were calculated with a two-sample *t*-test.

### 2.9. UDP-Glo Glycosyltransferase Assay

Next to CCEs, UDP-glycosyl transferases (UGTs) were well-represented in the differential gene expression analysis between bifenthrin-resistant and -susceptible strains. Among the differentially expressed UGTs, we identified *teturUGT29* (*tetur05g05060*) and *teturUGT10* (*tetur02g09830*), which were previously expressed by Xue et al. [54], originate from the strains ES1 and IT2, respectively, and were found to be able to glycosylate abamectin. In order to test whether these UGTs could also glycosylate bifenthrin or its metabolites (after cleavage of the ester bound), a UDP-Glo^TM^ glycosyltransferase assay (Promega, Madison, WI, USA) was performed, as described earlier [54,79]. In short, 50 μM bifenthrin (CAS number 82657-04-3, Sigma–Aldrich, Saint Louis, MO, USA), bifenthrin-alcohol (2-methyl-[1,1′-biphenyl]-3-yl)methanol, CAS number 76350-90-8, Sigma–Aldrich, Saint Louis, MO, USA) or TFP-acid (cis-3-(2-chloro-3,3,3-trifluoroprop-1-en-1-yl)-2,2-dimethylcyclopropanecarboxylic acid, CAS number 72748-35-7, Sigma–Aldrich, Saint Louis, MO, USA) (all substrates were solved in methanol), 0.1 μg enzyme (*teturUGT29* or *teturUGT10*) and 400 μM UDP-glucose were incubated in a total reaction volume of 125 μL containing 0.1 M sodium phosphate buffer (pH 7.5) and 16.7 mM MgCl_2_ at 25 °C for 1 h. The negative controls used for the calculations consisted of all reaction components except for the substrate (bifenthrin, bifenthrin-alcohol or TFP-acid). Additional negative controls were incorporated in the experiments, which contained all reaction components except for the enzyme or UDP-glucose. The formation of free-UDP in the glycosyltransferase reaction was quantified by the UDP-Glo^TM^ glycosyltransferase assay (Promega, Madison, WI, USA), as described in Snoeck et al. [79]. In this assay, the release of free-UDP is detected by the emission of light during a luciferase reaction in the conversion of free-UDP to ATP in a white, flat-bottom 96 (chimney)-well lumitrac medium binding assay plate (Greiner Bio-One). Luminescence was measured in Relative Luminescence Units (RLU) in triplicate with a Tecan infinet M200 plate reader (Tecan). A 0-25 µM free-UDP standard curve was plotted with SigmaPlot (version 14.5, Systat Software, San Jose, CA, USA) and used to calculate free-UDP. Statistical differences among substrates by UGT were assessed by analysis of variance (ANOVA), where significant differences were observed. Post-hoc Tukey tests were applied. Analyses were performed in R (version 4.1.2).

## 3. Results

### 3.1. Toxicity Bioassays

The susceptibility of bifenthrin and etofenprox was tested on ten *T. urticae* strains (Table 1). The LC_50_ values for bifenthrin ranged from 1.35 mg L^−1^ to more than 6000 mg L^−1^, while the LC_50_ values for etofenprox varied from 6.9 mg L^−1^ to 220 mg L^−1^. The red strains IT3, El Juan and SR6i and the green strains UK4 and RO1 were both susceptible to bifenthrin and etofenprox. The red strains MR-VL and ES1 and the green strain ROS-IT were highly resistant to bifenthrin and moderately resistant to etofenprox. The red strain ES1 was the most highly resistant to bifenthrin, as the RR exceeded 9000 when compared to the susceptible red strain IT3. The RRs of etofenprox were below 10 for all resistant strains, suggesting that there was only a slight cross resistance effect. The inbreeding of the MR-VL strain resulted in slightly lower bifenthrin resistance levels but a large increase in sensitivity towards etofenprox. Remarkably, the inbreeding of the strain ROS-IT resulted in a loss of resistance to bifenthrin, as well as to etofenprox.

### 3.2. Presence of Target-Site Mutations in the VGSC

To evaluate whether the resistance profile of the strains used in this study were associated with a known target-site mutation in the VGSC, available gene expression data (MR-VL, ES1, IT3, El Juan, UK4, RO1, ROS-IT) and DNA sequencing data (ROS-ITi, MR-VLi and SR6i) were screened for the presence of the voltage gated sodium channel mutations M918L, L925M, L1024V, F1534S and F1538I (Table 1). These target-site mutations were linked to pyrethroid resistance in previous studies [16,19,27,80]. Although the number of RNAseq read counts aligning with the VGSC gene was low for the strains El Juan, MR-VL, ES1, IT3, ROS-IT, UK4 and RO1, we observed the VGSC mutation L1024V in three out of four of the bifenthrin-resistant strains, fixed in MR-VL and MR-VLi, while they were present in 33% of the VGSC reads of ES1. Moreover, the highly bifenthrin-resistant ES1 also showed the presence of the F1538I target-site mutation in 25% of the reads. This target-site mutation was also observed in the susceptible strain UK4 (20% of the reads). None of the strains in this study had the M918L mutation. The L925M mutation was only found in one out of four reads in the bifenthrin-resistant ROS-IT, while its inbred derivative ROS-ITi was susceptible and apparently lost this mutation.

### 3.3. Bulked Segregant Analysis

#### 3.3.1. Resistance in Parental and Segregating Populations

**Figure 2:**
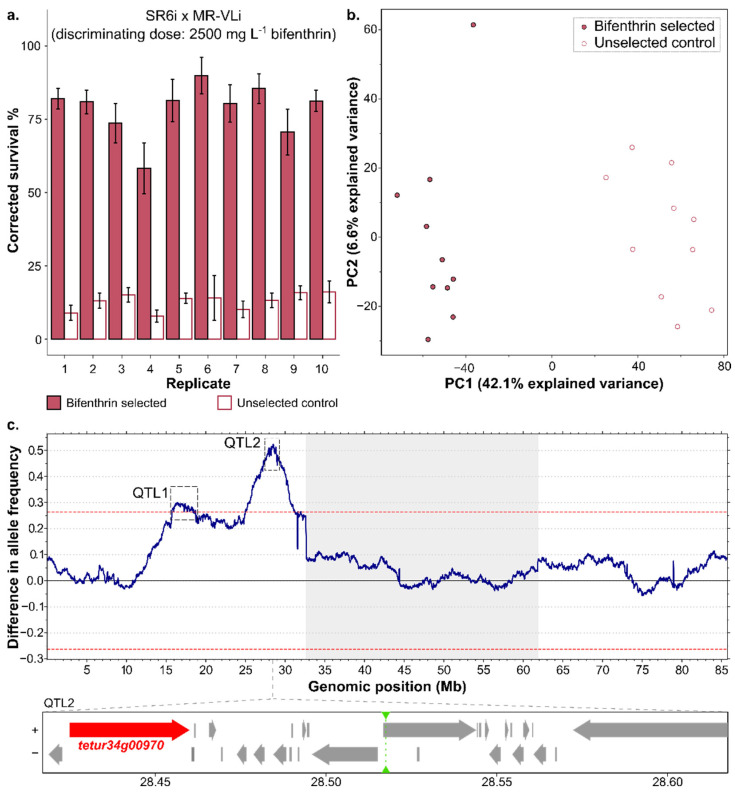
Bulked segregant analysis (BSA) to identify the genomic loci underlying bifenthrin resistance. (**a**) Adult survival of long-term selected and control (unselected) populations of SR6i x MR-VLi after acaricide application (2500 mg a.i. L^−1^ bifenthrin). The selected populations showed significantly higher survival rates compared to the control populations, showing that long-term bifenthrin selection results in bifenthrin resistance (F_1,69_ = 867.76, *p* < 2.2 × 10^−16^). Error bars represent the standard error of the mean. (**b**) Principal component analysis (PCA) of the unselected and bifenthrin-selected populations, based on genome-wide allele frequencies at polymorphic sites. PC1 clearly separates the selected populations from the unselected populations. (**c**) QTL mapping for bifenthrin resistance using a BSA. Averaged genome-wide differences in allele frequency using ten paired populations (bifenthrin-selected vs. unselected), with the dashed red lines delineating statistical significance for QTL detection (adjusted *p*-value (*p* adj) of 5%). Two QTLs on chromosome 1 exceed the 5% *p* adj threshold: QTL1 (16.2–17.4 Mb) and QTL2 (~28.5 Mb). Genes within 100 kb of QTL2 are depicted below the graph, and *tetur34g00970*, which encodes the VGSC, is highlighted in red. The location of the averaged BSA peak of QTL2 is indicated with a solid green triangle and a dashed line. The orientation of gene models is as indicated (“+” or “−” for forward and reverse strands, respectively).

Since previous studies not only reported the importance of VGSC mutations in pyrethroid resistance [16,17,18,19] but also reported other metabolic enzymes such as CCEs, GSTs and/or CYPs [17,43,44,45,46,48,49] to be involved, a bulked segregant analysis (BSA) was performed to unbiasedly identify the genomic loci involved in bifenthrin resistance. To facilitate the genetic mapping [81], available inbred strains were used—in particular, MR-VLi and SR6i (previously used in De Beer et al. [52]). MR-VL was selected because this strain and its inbred derivative MR-VLi were highly resistant to bifenthrin and displayed limited levels of cross-resistance towards etofenprox. In addition, previous studies also showed increased bifenthrin metabolism in the MR-VL strain [44,45]. To avoid the possible genetic incompatibilities that were previously observed when crossing red and green strains, we chose the red susceptible SR6i as the susceptible parent in the BSA. As shown in Table 1, the bifenthrin LC_50_ of MR-VLi still exceeded 2300 mg L^−1^, while the susceptible SR6i had an LC_50_ of 5 mg L^−1^. Females of the susceptible parent (SR6i) were crossed to males of the resistant parent (MR-VLi), and the population was allowed to expand for three generations, after which the LC_50_ was determined (Table S3). Next, this large segregating population was divided into ten subpopulations with bifenthrin selection and ten subpopulations without selection. After approximately 14 generations of experimental evolution, the acaricide-selected and control populations were phenotyped by exposure to a discriminating dose of 2500 mg L^−1^ bifenthrin. At this concentration, the bifenthrin-selected SR6i x MR-VLi populations had a significantly higher survival rate compared to their control populations (F_1,69_ = 867.76, *p* < 2.2 × 10^−16^, Figure 2), providing evidence that experimental evolution resulted in adaptation.

#### 3.3.2. Genomic Responses to Bifenthrin Selection

Genomic DNA was extracted and sequenced from each of the selected and unselected populations, and the genomic reads were aligned to the London reference genome [60]. A PCA revealed a clear separation between the replicates of the selected and unselected populations (Figure 2).

To reveal the genomic regions associated with bifenthrin selection, differences in allele frequencies between the selected and control populations were assessed as outlined in Kurlovs et al. [81]. Genome-wide allele frequencies between the bifenthrin-selected and unselected (control) populations (see gVCF/Data S1 at Figshare (https://doi.org/10.6084/m9.figshare.21446823.v1, accessed on 1 November 2022)) revealed large deviations in allele frequencies on chromosome 1 (Figure S1). With an adjusted *p*-value (*p* adj) of 5%, two significant peaks (QTL1 and QTL2) on chromosome 1 could be distinguished (Figure 2). QTL1 is a broad peak at 16.1–17.4 Mb and spans a region of more than 250 genes (Table S4), of which 7—3 GSTs and 4 ABC transporters (ABCs)—encoded members of enzyme or transporter families previously reported to be involved in the detoxification process (Table S5) [82]. In addition to detoxification enzymes and/or transporters, we also identified two nuclear receptors (E75, *tetur03g07550*, and HR3, *tetur03g08440*), which might play a role in regulating the gene expression of downstream detoxification genes, and a member (*tetur03g10113*) of the G-protein coupled receptor (GPCR) family, a family which has previously been implicated in the pyrethroid resistance of insects (reviewed in Amezian et al. [83]). Remarkably, 23 members of the chemosensory receptor family were also located in the QTL1 region. A voltage-gated calcium channel (VGCC, *tetur03g09070*) was also found in QTL1 and harbored a high impact mutation causing a frameshift. This mutation is fixed in MR-VLi and absent in SR6i, and the allelic depth of the variant allele (mutation) was higher than the allelic depth of the reference allele in all selected populations, while the allelic depth of the variant allele (mutation) was higher than that of the reference allele in only two control populations. The VGCC shows molecular similarities to the VGSC, and previous research showed an interaction between the VGCC and the pyrethroid deltamethrin, but not with bifenthrin [84,85].

In contrast to QTL1, QTL2 at ~28.5Mb is a sharp peak in which the VGSC gene (*tetur34g00970*), the target-site of bifenthrin, is located (Table S6). SNPeff analysis revealed that the L1024V mutation (*M. domestica* numbering) is fixed in the VGSC of the resistant parent MR-VLi and the bifenthrin-selected populations, whilst it is absent in the susceptible parent SR6i.

### 3.4. RNAseq Analysis

The BSA revealed that, next to target-site resistance, additional bifenthrin resistance mechanisms were most likely at play, as QTL1 contained diverse detoxification enzymes from multiple families and potential regulators of gene expression. To investigate these additional players, and to obtain a more global view on the gene expression changes associated with pyrethroids resistance, we performed a differential gene expression analysis on a panel of bifenthrin-resistant and -susceptible strains for which gene expression data were available (Table S7).

A principal component analysis (PCA) revealed that 35% of the total variation in gene expression could be explained by principal component 1 (PC1), while 16% was explained by PC2 (Figure S2). Further, the four red strains (ES1, IT3, El Juan and MR-VL) clustered closely together on PC1 and were separated from the green strains (UK4, RO1 and ROS-IT).

**Figure 3:**
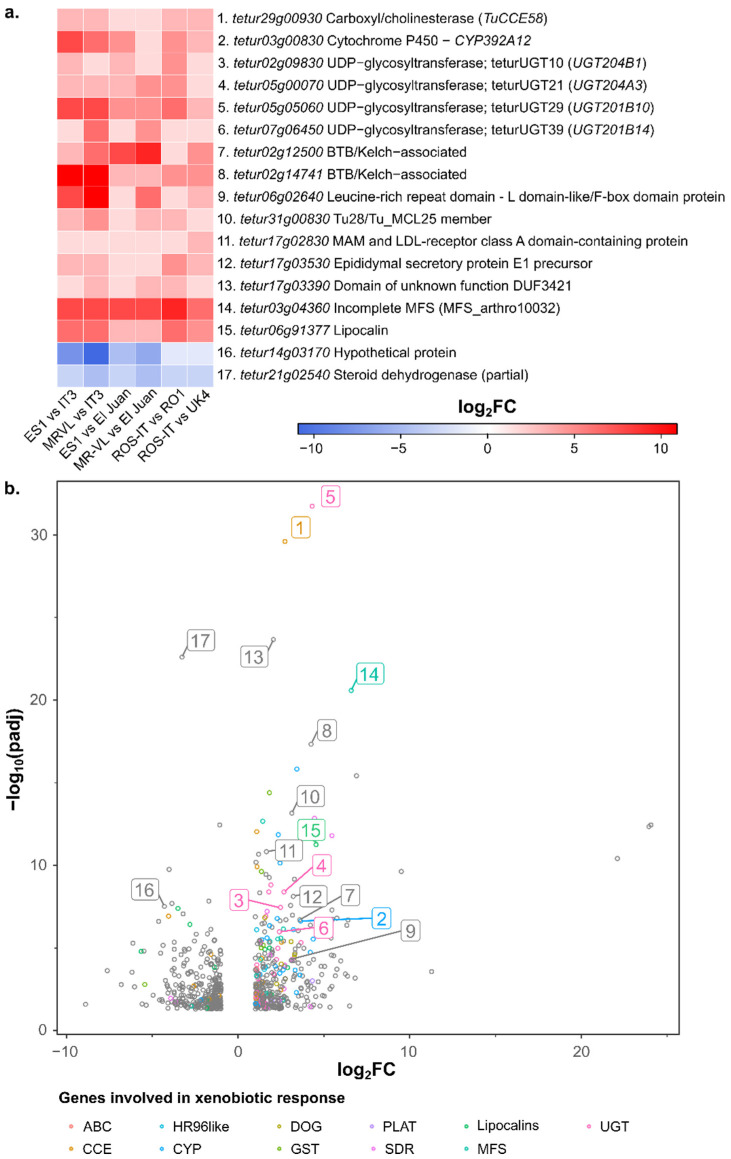
Differential gene expression analysis between bifenthrin-resistant and -susceptible strains. (**a**) Heatmap showing the differentially over- and underexpressed genes that are shared between the six pairwise comparisons (core set of differentially expressed genes). Numbers in the panel correspond to the numbers shown in the volcanoplot in panel b; (**b**) Volcanoplot showing the overexpressed and underexpressed genes (absolute log_2_FC ≥ 1 and *p* adj < 0.05) in the differential expression analysis between all bifenthrin-resistant and -susceptible *T. urticae* strains. Differentially expressed genes encoding detoxification enzymes (cytochrome P450 monooxygenases (CYPs), intradiol ring cleavage dioxygenases (DOGs), increased carboxyl/choline esterases (CCEs), glutathione S-transferase (GSTs), UDP-glycosyl transferases (UGTs)), transporters (ABC transporters (ABCs) and Major Facilitator Superfamily (MFS)), lipocalins, nuclear hormone receptor family 96 (HR96) lacking DNA binding domain (HR96like) [66], short-chain dehydrogenases/reductases (SDRs) or PLAT domain proteins (PLAT) are color-coded according to the legend shown below the plot.

Differential gene expression analysis was performed by pairwise comparisons between bifenthrin-resistant and -susceptible non-inbred *T. urticae* strains (green strain comparisons: ROS-IT vs. UK4 and ROS-IT vs. RO1; red strain comparisons: ES1 vs. IT3, MR-VL vs. IT3, ES1 vs. El Juan, MR-VL vs. El Juan) (absolute log_2_FC ≥ 1 and an adjusted *p*-value (*p* adj) < 0.05; a complete list of differentially expressed genes for each pairwise comparison can be found in Table S8.1-6 and is shown in Figure S3). The core set of differentially expressed genes shared between all pairwise comparisons (absolute log_2_FC ≥ 1 and *p* adj < 0.05 in each of the six comparisons) consisted of 17 genes (Table S9) and is shown in Figure 3. We also performed an alternative differential expression analysis based on the comparison between all the bifenthrin-resistant strains (ES1, MR-VL and ROS-IT) and all the bifenthrin-susceptible strains (IT3, El Juan, RO1 and UK4) regardless of color morphotype. This resulted in 352 over- and 309 underexpressed genes (absolute log_2_FC ≥ 1 and *p* adj < 0.05) (Table S8.7_DGE), which included the core set of differentially expressed genes from the first analysis (Figure 3), providing further support for their importance. Notably, although not located in a QTL region of the BSA analysis, the detoxification enzyme and/or transporter genes *tetur29g00930* (*CCE58*), *tetur03g00830* (*CYP392A12*), *tetur02g09830* (*teturUGT10*), *tetur05g00070* (*teturUGT21*), *tetur05g05060* (*teturUGT29*), *tetur07g06450* (*teturUGT39*) and *tetur03g04360* (incomplete MFS) were considered as resistance gene candidates based on their expression patterns.

Comparing the differential gene expression analyses with the genes covered in the BSA QTL1 region (16.1–17.4 Mb, Table S4) showed that, of the seven genes encoding members of detoxification enzymes or transporters found in QTL1, only TuABCC-08, (*tetur03g07490)*, TuABCC-07 (*tetur03g07460*) and TuGSTd06 (*tetur03g07920*) were significantly overexpressed by two-fold or more, and one incomplete GST, TuGSTinc03 (*tetur03g07880*), was significantly underexpressed in one or more pairwise comparisons between a bifenthrin-resistant and -susceptible strain. Moreover, the nuclear receptor E75 (*tetur03g07550*) in QTL1 was significantly overexpressed in three out of the six pairwise differential gene expression comparisons and in the alternative differential expression analysis comparing all resistant to all susceptible strains (log_2_FC of 3.5). Additionally, the G-protein coupled receptor (GPCR, *tetur03g10113*) in QTL1 shows overexpression in two out of six pairwise comparisons. On the contrary, HR3 found in QTL1 was significantly underexpressed in two pairwise comparisons and in the alternative differential gene expression method (log_2_FC of -2).

### 3.5. Bifenthrin Metabolizing Activity of Recombinantly Expressed CCE58 and CCEinc18

Since previous research with the bifenthrin-resistant strain MR-VL showed increased bifenthrin-hydrolyzing activity in MR-VL compared to bifenthrin-susceptible strains [44,45], we focused on CCEs in the differential gene expression analysis. The genes *CCE58* and *CCEinc18* were overexpressed in pairwise differential gene expression comparisons and/or in the differential expression analysis comparing all bifenthrin-resistant strains to all bifenthrin-susceptible strains (Tables S8 and S9). A BLASTn search against the de novo transcriptome assembly of MR-VL (File S1), using *CCEinc18* and *CCE58* of the London genome [60] as a query, yielded the best hit with contig 6457 (97.4% identity) and contig 1813 (97.7%), respectively. Based on this best match and on previously published genomic data, we found that the MR-VL allele of *CCEinc18* was a full-length coding gene, in contrast to the reference London genome. Next, the CCE genes were codon-optimized (File S2) and functionally expressed in a *P. pastoris* expression system to determine the bifenthrin metabolizing activity of these enzymes. As shown in Figure S4, both CCEs are abundantly expressed by recombinant *P. pastoris* colonies and successfully purified using the Ni-NTA columns, resulting in a single band at ±75 kDa on both SDS-PAGE and Western blot.

The activity of the recombinantly expressed CCEs, CCE58 and CCEinc18 was determined against two model substrates (1-NA and 4-NPA). The kinetic parameters (V_max_, K_m_) are represented in Table 2. The maximum velocity (V_max_) is slightly higher for CCEinc18 for both 1-NA and 4-NPA. Additionally, the Michaelis–Menten constant (K_m_) of CCEinc18 is higher than that of CCE58 for the substrate 4-NPA. However, for 1-NA, K_m_ is lower for CCEinc18 than for CCE58, pointing towards a higher affinity of CCEinc18 for 4-NPA and of CCE58 for 1-NA.

**Figure 4:**
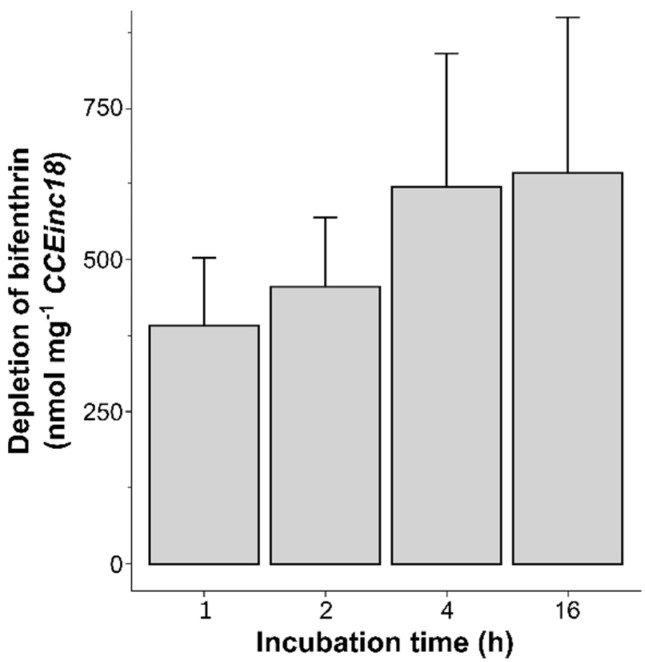
Bar chart showing the amount of metabolized bifenthrin by the recombinantly expressed MR-VL CCEinc18 after 1, 2, 4 and 16 h of incubation.

Given its potential involvement in bifenthrin resistance, the ability of both CCEs (CCE58 and CCEinc18) to metabolize bifenthrin was investigated by in vitro incubations followed by gas chromatography quantification. For each experiment, the peak areas detected at a retention time of 17.24 min were plotted against a technical calibration curve of bifenthrin (0.1–4 ppm) in order to link the peak area to a remaining bifenthrin concentration (ppm) for each sample. The bifenthrin depletion rates for active and inactivated CCE58 and CCEinc18 proteins are shown in Table 3. Actual bifenthrin depletion rates for each protein are calculated by the subtraction of the bifenthrin depletion in the inactive protein samples from that of the active protein samples. For CCEinc18, there was an average bifenthrin depletion of 19.58%, with a significant corrected bifenthrin depletion rate (mean ± SD) of 3.5 ± 1.1 nmol min^−1^ mg^−1^ protein. This is almost 20-fold higher than the corrected bifenthrin depletion rate of CCE58, where there is, on average, only 1.09% bifenthrin depletion with a corrected bifenthrin depletion rate (mean ± SD) of 0.20 ± 0.59 nmol min^−1^ mg^−1^ protein, which is not significantly different from the other treatments. Notably, for both proteins, the bifenthrin depletion rate of the inactivated protein is not significantly different from the samples without supplemented protein. Given the potential capability of CCEinc18 to metabolize bifenthrin, as indirectly indicated by bifenthrin depletion, a time curve of bifenthrin depletion after 1, 2, 4 and 16 h of incubation with CCEinc18 was determined and is represented in Figure 4. As there were no significant differences in bifenthrin depletion upon incubation with boiled protein versus buffer control, as shown in Table 3, only controls without protein were analyzed. Despite the relatively large error bars, the amount of depleted bifenthrin by CCEinc18 clearly increased from 1–4 h of incubation, whereas, from 4 h to 16 h, the amount of depleted bifenthrin increased less dramatically.

### 3.6. Glycosylation Assay

The ability of teturUGT29 and teturUGT10 to glycosylate bifenthrin and its metabolites was evaluated using a glycosylation assay. These two *T. urticae* UGTs were selected based on the differential expression analysis between bifenthrin-resistant and -susceptible strains and on the availability of functionally expressed UGTs [54,79]. The chosen UGTs, teturUGT29 and teturUGT10, belong to different UGT families, UGT201 and UGT204, respectively [79], and originate from the strains ES1 and ROS-IT, both being more than 1000-fold resistant to bifenthrin, respectively [54]. The substrate preference of the selected *T. urticae* UGTs was evaluated with bifenthrin and bifenthrin’s metabolites after the cleavage of the ester bound (TFP-acid and bifenthrin-alcohol) using the UDP-Glo^TM^ glycosyltransferase assay (Promega, Madison, WI, USA). Free-UDP was calculated from a 0–25µM free-UDP standard curve (Figure S5). Assays with bifenthrin and the enzyme teturUGT29, and TFP-acid and teturUGT10 caused very high background values (RLU) in the negative control (solvent without substrate), resulting in a negative free-UDP value. TeturUGT29 showed a very low activity towards the three substrates (Table 4), while teturUGT10 exhibited moderate to high activity towards bifenthrin and bifenthrin-alcohol, respectively. The high free-UDP value (±21 µM) observed when incubating the substrate bifenthrin-alcohol with the enzyme teturUGT10 indicated that bifenthrin-alcohol is the preferred substrate out of the tested substrates.

## 4. Discussion

The VGSC is the target-site of pyrethroids, and reduced target-site sensitivity is known to be one of the major mechanisms involved in pyrethroid resistance [1]. Nevertheless, the increased metabolism of pyrethroids by detoxification enzymes, such as CCEs, CYPs, GSTs and UGTs, has also been implicated in pyrethroid resistance in many insect and mite species. For example, glycoside conjugation mediated by a UGT has been associated with pyrethroid resistance in *Anopheles sinensis* [86]. For *Nilaparvata lugens*, GST-amplification has been linked with pyrethroid resistance [87], while in a resistant strain of *P. citri*, GSTs also seemed to play an antioxidant role in the oxidative stress caused by the pyrethroid fenpropathrin [88]. On the other hand, the CYPs of mosquitoes are well known to metabolize pyrethroids and were reported to be overexpressed in many pyrethroid-resistant strains (reviewed in David et al. [89]). For the house fly *M. domestica*, Feng et al. (2018) showed that both the constitutive and inductive overexpression of CCEs resulted in the enhanced detoxification of pyrethroids [90]. Previously, our research group and colleagues showed that both target-site insensitivity and CCEs were involved in bifenthrin resistance in *T. urticae* populations [17,19,44,45]. In this study, using a collection of resistant and susceptible strains, and including unbiased genetic approaches to resolve resistance mechanisms, we further investigated the bifenthrin resistance mechanisms in *T. urticae* in depth.

First, we performed toxicity assays with the Type I ester pyrethroid bifenthrin and a non-ester pyrethroid etofenprox on a panel of *T. urticae* strains. To examine whether the difference in susceptibility to bifenthrin could be explained by target-site resistance mutations, we screened for known mutations in the VGSC and found that three out of four bifenthrin-resistant strains carried the L1024V mutation in the VGSC (MR-VL, MR-VLi and ES1). Next to the L1024V mutation, the most resistant strain in this study, ES1, also showed the presence of the F1538I mutation, which has been frequently associated with pyrethroid resistance [16,17,18,19,30,91,92,93,94]. We did find the F1538I mutation in the VGSC of the susceptible strain UK4, but it was at a low frequency and therefore probably did not result in resistance in our bioassays. Tan et al. (2005) showed with site-directed mutagenesis and functional expression in *Xenopus laevis* oocytes that the F1538I substitution causes the insensitivity of the VGSC to several Type I and Type II pyrethroids, although bifenthrin was not included in Tan’s study [95]. Further, the presence of an L925M mutation in the VGSC of the highly bifenthrin-resistant strain ROS-IT and its loss in its susceptible derivative inbred line indicated the importance of the L925M mutation in bifenthrin resistance. Very recently, this mutation was also reported for bifenthrin-resistant *T. urticae* strains in a study investigating gene regulation mechanisms [20]. Notably, amino acid substitutions at this VGSC position—leucine to valine (L925V), methionine (L925M) or isoleucine (L925I)—have also been correlated with pyrethroid resistance in other mites and ticks, including the *Varroa* mite, the poultry red mite *D. gallinae*, predatory mites such as *P. persimilis* [26,27,29,80], the cattle tick *Rhipicephalus microplus* and insects such as the common bedbug *Cimex lectularius* and several species of whiteflies [10]. The presence of different target-site resistance mutations also reveals an independent origin of resistance in the different strains [96].

The resistance ratios for etofenprox were much lower compared to those of bifenthrin, which is in line with previous studies with *A. gambiae* [97,98], where toxicity tests revealed a high resistance to the VGSC modulators permethrin (Type I), deltamethrin (Type II) and DDT but not to etofenprox. The difference in molecular structure between non-ester (etofenprox) and ester (bifenthrin) pyrethroids may affect the interaction with the target-site, and, consequently, target-site mutations may contribute to the resistance phenotype to a different extent. In 2017, Riga et al. investigated the specific contribution of target-site resistance mutations to Type I (bifenthrin) and Type II (fenpropathrin and fluvalinate) pyrethroid resistance via the marker-assisted back crossing of these mutations in an independent susceptible genetic background. This study revealed that resistance to fenpropathrin and fluvalinate could be completely explained by target-site mutations, while these mutations could only partly explain the bifenthrin resistance phenotype [19]. Likewise, Hu et al. (2011) investigated the effect of a phenylalanine to cysteine mutation (F1515C) in the VGSC of *Aedes* sp. and found a reduced sensitivity of the mutated channels to Type I pyrethroids but did not observe an effect with Type II pyrethroids [99]. Such pyrethroid-specific sodium channel mutations were also described by Du et al. (2009) [100], while in 2012, Schleier and Peterson observed that the combination of the non-ester pyrethroid etofenprox with Type I or Type II pyrethroids differed in toxicity towards *Drosophila melanogaster* [13], with the combination of permethrin (Type I) + etofenprox having antagonistic toxicity, while the combination of cypermethrin (Type II) + etofenprox was synergistic, suggesting a common binding site for the latter two compounds.

Our toxicity tests revealed a different susceptibility towards bifenthrin between strains with the same target-site mutations and large differences between etofenprox and bifenthrin resistance ratios, possibly pointing towards an increased CCE-mediated metabolism of bifenthrin. In addition, since bifenthrin resistance could only be partially explained by target-site resistance in *Tetranychus urticae* [19], and previous studies, including a study with the strain MR-VL, already suggested an increased detoxification [17,43,44,45,46,48,49], we performed a bulked segregant analysis (BSA) to identify the genomic loci associated with bifenthrin resistance in the strain MR-VLi, without any prior hypothesis. Two genomic loci responded to the long-term selection with bifenthrin: QTL1 at 16.1–17.4 Mb and QTL2 at 28.5 Mb. The sharp peak of QTL2 is in close proximity (±60kb) to the gene *tetur34g00970*, encoding the VGSC. Within this target-site gene, we identified a fixed L1024V mutation in the resistant parental strain and in the selected populations of the BSA experimental set-up. We also identified a second QTL in our BSA genetic mapping experiment, which was a broad peak covering a large region of the *T. urticae* genome and contained—among a variety of other genes—a VGCC. Despite the VGCC and VGSC being molecularly very similar, and the shown affinity of a pyrethroid insecticide (deltamethrin) for the VGCC [84], a previous study also showed that the VGCC only interacts with pyrethroids possessing an α-cyano group [85]. Therefore, it is unclear whether the VGCC is involved in bifenthrin resistance in *T. urticae*.

However, we could not pinpoint an exact resistance candidate gene for QTL1. The presence of this QTL strongly suggested that a secondary resistance mechanism was at play in the bifenthrin-resistant strain MR-VLi. To gain more insight into possible metabolic players involved in bifenthrin metabolism, a differential gene expression analysis between bifenthrin-resistant and -susceptible strains was performed. This showed that three genes encoding members of detoxification enzymes or transporters (TuABCC-08, TuABCC-07 and TuGSTd06)—and present in QTL1—were significantly overexpressed by two-fold or more in one or more of the pairwise differential gene expression analyses. Although ABCs and GSTs were previously shown to be associated with pyrethroid resistance, [35,87,88,101,102,103], we could not technically validate the role of ABC transporters in this study.

Instead, our focus was on genes belonging to the CCE and UGT gene families—in particular, *CCE58*, *teturUGT10*, *teturUGT21*, *teturUGT29* and *teturUGT39*, which were overexpressed in all pairwise comparisons between bifenthrin-susceptible and -resistant strains. As described by Yang et al., (2002) and Ay and Gurkan (2005) [43,49], bifenthrin resistance in *T. urticae* was correlated with increased esterase activity. Additionally, Van Leeuwen et al. showed that the esterase activity in MR-VL, the parental strain of the inbred strain used in our BSA experiment, was higher compared to a laboratory susceptible strain [44,45], and the susceptibility towards bifenthrin was synergized significantly with the esterase inhibitor DEF. Previous studies have shown that CCEs could metabolize pyrethroids, including bifenthrin. Shi et al. (2016) showed that the esterase *TCE2* of *T. cinnabarinus* (Boisduval) was able to degrade the pyrethroid fenpropathrin [104], while, very recently, it was also shown that an esterase of the fungus gnat, *Bradysia odoriphaga*, had significant hydrolase activity towards the ester-containing substances bifenthrin and malathion [105]. Lastly, Li et al. reported that two esterases of *Plutella xylostella* (Pxα14 and PxEST-6) could metabolize bifenthrin and that the RNAi knockdown of Pxα14 caused an increase in resistance to bifenthrin [106,107]. Based on these examples, we selected two CCEs—CCE58, overexpressed in all pairwise comparisons, and CCEinc18, overexpressed in four pairwise differential gene expression analyses—as the top candidates to study their bifenthrin-metabolizing potential. Both CCE58 and CCEinc18 were recombinantly expressed and active, but only CCEinc18 was able to significantly deplete bifenthrin levels in the assay, with the highest activity being within the first hours of incubation. This confirmed the findings of Van Leeuwen et al. [44,45], who showed that bifenthrin was a substrate for CCEs with an overall higher metabolism of bifenthrin in the resistant *T. urticae* strain MR-VL. Nevertheless, while we show clear bifenthrin depletion only in the presence of active enzymes in our assays, we did not identify nor quantify the metabolites, and, thus, formal evidence of metabolism is still lacking. Lastly, given the overall slow depletion rates detected, a role of both CCEs in resistance via sequestration should not be ruled out. Indeed, binding followed by very slow metabolism is a well-known resistance mechanism associated with CCEs [108].

Although various evidence points towards an important role of CCEs in bifenthrin resistance, our differential gene expression analysis also showed that UGTs were overexpressed in resistant strains and might be involved in bifenthrin resistance. Notably, a higher UGT activity was also observed in a highly bifenthrin-resistant strain from China (JY-HN, with an RR >500 fold), in which the F1538I mutation was present at a low frequency [92]. In this study, teturUGT10 and teturUGT29, enzymes from ES1 and IT2 [54], were tested in a glycosyltransferase assay, and we found that teturUGT10 could glycosylate both bifenthrin and the bifenthrin-alcohol metabolite. This would imply that a part of bifenthrin spontaneously hydrolyzes to bifenthrin-alcohol in our experiment, as UGTs preferentially glycosylate substrates containing a nucleophilic O-, N- or S-group [109]. However, this spontaneous hydrolysis is rare, as bifenthrin is reported to be stable to aqueous hydrolysis and photolysis [110,111,112], and we cannot readily explain this observation. It is also not clear whether the bifenthrin-alcohol is still toxic [7] and, therefore, whether UGT-mediated glycosylation contributes to bifenthrin resistance. At least in vivo, bifenthrin might first be hydroxylated to 4-OH bifenthrin by a CYP, providing the nucleophilic group for glycosylation and subsequent further excretion. Unfortunately, we were not able to test this hypothesis, as 4-OH bifenthrin was not commercially available. However, this metabolic pathway (CYP hydroxylation followed by UGT-conjugation) has been previously observed by Ji et al. (2021) for fish [113] and has been considered the main metabolic pathways of bifenthrin in vertebrates, next to esterase-mediated hydrolysis [114]. Although previous research on the bifenthrin-resistant MR-VL strain did not show PBO synergism [45], evidence towards the involvement of CYPs was given for a different *T. urticae* strain by showing the increased susceptibility towards bifenthrin after the RNAi silencing of cytochrome P450 reductase (CPR), an essential enzyme that serves as an electron donor for CYPs [115]. However, to what extent this was due to a general fitness effect, as CPR regulates many endogenous P450 essential reactions, or a specific P450 hydroxylation in detoxification, is not clear. Future research on *T. urticae* aiming at unravelling the potential role of CYPs in bifenthrin resistance could focus on the transformation of bifenthrin into 4-OH bifenthrin in *T. urticae*’s microsomal fraction, as previously investigated by Zimmer and Nauen (2011) with deltamethrin in *Meligethes aeneus* [116]. Notably, we found that *CYP392A12*—belonging to our core set of overexpressed genes in the differential expression analyses—was previously found to be strongly associated with cyenopyrafen resistance [117]. Unfortunately, our research group was not able to functionally express CYP392A12 despite several optimization efforts [117], and we were not able to characterize this CYP in terms of bifenthrin metabolism.

Multiple detoxification genes were overexpressed in resistant *T. urticae* strains, but genes encoding these enzymes were not located within QTL1 of QTL2 in our QTL mapping experiment. This might suggest that the expression of these genes is regulated by a *trans* acting factor. Recently, such *trans*-driven regulation of detoxification genes has been clearly demonstrated for *T. urticae* [20], and therefore, we also examined QTL1 for transcription factors. In the QTL1 region [20], we found an E75 nuclear receptor which was significantly upregulated (log_2_FC of 3.51) in the differential gene expression analysis comparing all resistant to all susceptible strains. In *Drosophila*, the nuclear receptor E75 harbors a heme group in its ligand-binding domain which can bind small gas molecules (nitric oxide and carbon monoxide) [118,119], but, to our knowledge, a role in the regulation of detoxification enzymes has not yet been reported. Lastly, within QTL1, we also found multiple chemosensory receptor (CR) genes. With the identification of over 400 intact gustatory receptors in *T. urticae* and CRs playing an important role in host plant acceptance in arthropods [120,121], one can speculate that direct (binding) or indirect interactions of bifenthrin with CRs might lead to the activation of detoxification pathways, and, hence, CRs might function as xenobiotic sensors [122].

## 5. Conclusions

In this study, we investigated bifenthrin resistance in the spider mite *T. urticae*. By performing a bulked segregant analysis, two genomic loci on chromosome 1 (QTL1 and QTL2) underlying bifenthrin resistance were identified. While we were not able to pinpoint possible resistance mechanisms to QTL1, we identified the target-site of pyrethroids, the VGSC, at QTL2. Moreover, the well-known L1024V mutation was present in the VGSC of the resistant parental strain and the selected BSA populations. Next to this VGSC mutation, the L925M and F1538I mutations were also associated with bifenthrin resistance by screening several bifenthrin-resistant strains. Since QTL1 was indicative of additional players in the bifenthrin resistance phenotype, we screened for these additional mechanisms in a differential gene expression analysis between several bifenthrin-resistant and -susceptible spider mite strains and found several overexpressed genes encoding detoxification enzymes, including UGTs, CCEs and a CYP. The roles of a number of those enzymes were functionally validated, with CCEin18 being able to metabolize bifenthrin, while teturUGT10 could glycosylate bifenthrin and bifenthrin-alcohol. It can be concluded that *T. urticae*’s resistance towards bifenthrin is caused by a complex interplay between toxicodynamic and toxicokinetic mechanisms.

## Figures and Tables

**Table 1:** LC_50_ values for the pyrethroids bifenthrin and etofenprox, tested on several *T. urticae* strains, with resistance ratios (RR) calculated relative to IT3 and RO1 in the red and green strains, respectively, and the percentage of reads that align with the voltage-gated sodium channel (VGSC, *tetur35g00970*) and harbor resistance mutations. (*n* = number of mites, LC_50_ = lethal concentration for 50% of the subjects, df = degrees of freedom, SE = standard error, CI = confidence interval, RR = resistance ratio, df = degrees of freedom.)

	Bifenthrin					Etofenprox					VGSC Mutations				
Strain	n	χ² (df)	Slope (±SE)	LC_50_ (95% CI)	RR (95%CI)	n	χ² (df)	Slope (±SE)	LC_50_ (95% CI)	RR (95% CI)	M918L	L925M	L1024V	F1534S	F1538I
El Juan	490	43.27 (18)	1.82 (±0.19)	2.9 (2.0–3.9)	2.2 ^a^ (1.4–3.4)	585	54.58 (18)	3.13 (±0.46)	24 (17–30)	1.02 ^a^ (0.84–1.2)	-	-	-	-	-
SR6i	1120	81.58 (38)	1.76 (±0.12)	5.4 (4.4–6.4)	4.0 ^a^ (2.6–6.2)	520	8.74 (18)	2.61 (±0.24)	6.9 (5.9–7.9)	0.30 ^a^ (0.24–0.37)	-	-	-	-	-
MR-VL	506	18.65 (18)	2.08 (±0.31)	3400 (2700–4600)	2500 ^a^ (1500–4100)	479	55.26 (18)	3.36 (±0.34)	220 (170–270)	9.4 ^a^ (7.8–11)	-	-	100%	-	-
MR-VLi	727	53.83 (26)	1.59 (±0.17)	2400 (1800–3600)	1800 ^a^ (1100–2900)	445	26.83 (18)	2.19 (±0.21)	60 (47 -74)	2.6 ^a^ (2.1–3.2)	-	-	100%	-	-
ES1	1082	59.18 (38)	2.06 (±0.48)	6300 (4200–22,000)	9500 ^a^ (2500–35,000)	512	26.79 (18)	4.00 (±0.71)	190 (120–240)	8.3 ^a^ (6.4–11)	-	-	33%	-	25%
IT3	446	24.77 (18)	0.94 (±0.16)	1.35 (0.62–2.1)	/	479	14.02 (18)	2.60 (±0.23)	23 (20–26)	/	-	-	-	-	-
ROS-IT	950	28.09 (38)	0.678 (±0.081)	3500 (2100–7200)	1470 ^b^ (770–2800)	542	8.31 (18)	2.59 (±0.71)	93 (20–150)	9.0 ^b^ (4.2–19)	-	25%	-	-	-
ROS-ITi	482	26.74 (21)	2.02 (±0.22)	3.2 (2.4–4.1)	1.4 ^b^ (1.0–1.8)	536	47.89 (21)	3.03 (±0.36)	12.5 (9.3–15)	1.2 ^b^ (1.0–1.5)	-	-	-	-	-
UK4	1056	70.27 (38)	1.397 (±0.099)	5.5 (4.0–7.0)	2.3 ^b^ (1.7–3.1)	458	19.98 (18)	2.99 (±0.27)	21 (18–24)	2.0 ^b^ (1.7–2.4)	-	-	-	-	20%
RO1	911	52.23 (38)	1.65 (±0.13)	2.4 (1.7–3.0)	/	426	43.21 (18)	4.23 (±0.45)	10.3 (8.3–12)	/	-	-	-	-	-

^a^ RR and corresponding 95% CI are calculated relative to the LC_50_ of IT3; ^b^ RR and corresponding 95% CI are calculated relative to the LC_50_ of RO1. Red strains are underlined.

**Table 2:** Kinetic parameters of CCE reference substrates determined with 1 µg protein of the recombinantly expressed MR-VL CCE58 and CCEinc18.

Reference Substrate	CCE58		CCEinc18	
	V_max_ (±SE)	K_m_ (±SE)	V_max_ (±SE)	K_m_ (±SE)
4-Nitrophenyl acetate ^a^	76.2 (±2.2)	0.379 (±0.028)	92.0 (±4.6)	0.633 (±0.071)
1-Naphthyl acetate ^b^	48.6 (±2.2)	0.446 (±0.051)	62.7 (±2.6)	0.325 (±0.038)

^a^ V_max_ and K_m_ expressed as nmol 4-nitrophenol min^−1^ mg^−1^ protein and mM, respectively. ^b^ V_max_ and Km expressed as nmol 1-naphthyl min^−1^ mg^−1^ protein and mM, respectively.

**Table 3:** Bifenthrin depletion rates of active and inactivated MR-VL CCE58 and CCEinc18.

Protein	Activity	Bifenthrin Depletion Rate ^a^ (Mean ±SE)	Corrected Bifenthrin Depletion Rate ^b^ (Mean ±SE)
CCE58	Active	0.78 ± 0.78	0.20 ± 0.59
	Inactivated	0.58 ± 0.43	
CCEinc18	Active	3.79 ± 0.95 *	3.5 ± 1.1 *
	Inactivated	0.29 ± 0.24	

^a^ nmol bifenthrin depletion mg^−1^ protein min^−1^ compared to the control without protein. ^b^ relative nmol bifenthrin metabolization in mg^−1^ protein min^−1^, as calculated by subtracting the inactivated bifenthrin depletion rate from the active bifenthrin depletion rate. * indicates a significant (*p* adj < 0.05) difference between the means of the bifenthrin control samples (without protein supplementation) and the protein-spiked samples, as determined by a two-sample *t*-test.

**Table 4:** Glycosylation characteristics of teturUGT29 and teturUGT10 in combination with bifenthrin, TFP-acid and bifenthrin-alcohol.

Substrate	Free-UDP ± SE (µM)	
	teturUGT29 (*tetur05g05060*)	teturUGT10 (*tetur02g09830*)
Bifenthrin	−0.76 ± 0.21	5.1 ± 3.4
TFP-acid	0.55 ± 0.23	−2.5 ± 3.7
Bifenthrin-alcohol	0.924 ± 0.020	21.1 ± 2.1 *

* indicates a significant (*p* adj < 0.05) difference between the mean of bifenthrin-alcohol and the means of TFP-acid and bifenthrin in combination with teturUGT10, as determined by one-way ANOVA with post-hoc Tukey.

## Data Availability

DNA and RNA-seq read data have been deposited to NCBI (PRJNA895330, PRJNA896577). Pairwise strain variant call data and read count data have been deposited to Figshare (https://doi.org/10.6084/m9.figshare.21446823.v1, accessed on 1 November 2022). Additional data are provided in the supplementary tables and files.

## Supplementary Materials

The following supporting information can be downloaded at: https://www.mdpi.com/article/10.3390/biology11111630/s1, Figure S1: Deviations in allele frequencies of bifenthrin (BIF)-selected and unselected (control, CON) populations of the bulked segregant analysis; Figure S2: Principal component analysis of the RNAseq expression data of the green strains ROS-IT, UK4 and RO1 and the red strains El Juan, MR-VL, ES1 and IT3; Figure S3: Venn diagrams showing the over- and underexpressed genes with absolute log_2_FC ≥ 1 and *p* adj < 0.05 in the pairwise comparisons between the susceptible and resistant *T. urticae* strains; Figure S4: Stain free SDS-PAGE (left panel) and Western blot (right panel) of CCE58 and CCEinc18 along several steps in the expression purification process; Figure S5: Free-UDP standard curve with Relative Luminescence Units (RLU) as a function of free-UDP (range of 0–25 µM), measured with a UDP-Glo^TM^ Glycosyltransferase Assay (Promega, Madison, Wisconsin, USA); Table S1: Target-site resistance mutations associated with Type I and Type II pyrethroids; Table S2: Composition of media used for the recombinant expression of *CCE58* and *CCEinc18* in *Pichia pastoris*; Table S3: Toxicity bioassays with bifenthrin on segregating crosses SR6i x MR-VLi and parental strains SR6i, MR-VLi; Table S4: List of genes covered by BSA’s QTL1 (Chr. 1; 16.1–17.4Mb); Table S5: List of detoxification genes used for volcanoplot in Figure 3; Table S6: List of genes covered by BSA’s QTL2 (Chr. 1, 28,5 Mb ±500kb); Table S7: Information of selected populations used for differential expression analysis; Table S8: Differentially expressed genes (DEGs) and significantly over/underexpressed genes between bifenthrin-resistant populations (ROS-IT, ES1 and MR-VL) and bifenthrin-susceptible populations (UK4, RO1, IT3 and El Juan) of *T. urticae*; Table S9: Core set of differentially expressed genes; File S1: Original nucleotide sequences of *CCE58* and *CCEinc18* in MR-VL; File S2: Codon-optimized nucleotide sequences of *CCE58* and *CCEinc18*.
