# Supplementary material for: High-Resolution Genetic Mapping Combined with Transcriptome Profiling Reveals That Both Target-Site Resistance and Increased Detoxification Confer Resistance to the Pyrethroid Bifenthrin in the Spider Mite Tetranychus urticae
Source: Biology (Basel). 2022 Nov 7;11(11):1630. doi: 10.3390/biology11111630 (PMC9687926; doi:10.3390/biology11111630)
Supplement: Supplementary file 1 [file biology-11-01630-s001.zip › FileS2_OptimizedSequencesEsterases.pdf]

Codon optimized sequence of CCE58

GAATTCAAAATCGTCGAGCCAGAGTTGACATTGAGTTCAGGTTCCATTAGAGGTACTTCCGTTGACTTCAGAGGAGTTAAGGT
TTATCAGTTTTTGGGTATTCCTTTTGCTGAACCACCTTTGAATGAGTTGAGATTCCAAAAGCCAGTTCCTAAGAAACCATGGA
ACGGTGTTTTGTCTGTTAACAAGTGGGGTTCTGCTTGTATGCAACCTGTTTTTCCAGGTTTCAACACTGAATTGCATTTGTCT
GAGGATTGTTTGATCTTGAACGTTTTCACTACTGAAGCTGCTTTCCAAGATAAGCAAAACGGTAAAAAGCATAAGCTTAGACC
AGTTATGGTTTGGATTCACGGTGGTTCTTTTAACTACGGTTCTGCTAATACTCCTGATCAACACGATGGTACTCCAATCACTG
GTTTGAAGGATGTTATCATCGTTTCTATTAATTACAGATTGGGTTCTTTGGGTTTCTTGCATTTGCCAGAAGCTGGTGTTCAC
GGTAACATGGGTTTGTGGGATCAACAATTGGCTTTGAAGTGGGTTAAGGATAACATCGAACATTTCGGTGGAGATCCAAATAA
GGTTACTATTTTCGGAGAGTCTGCTGGTTCTATGTCTGTTTCTGCTCACATTGTTTCTCCACAATCTAAGGGTTTGTTTACTA
ACGCTATCATGCAATCTGGTTCTATCTACGATTTGGATAGATGGACTCAACCAGGTTTGGTTGCTAACTTCACTTCTAAGATC
GGTTGTATCTCTGATAACTACAAGTCTTGTTTGTCTAACTACAGATTTGGTCAATTCCCTGAAGCTGATAGATTGACTTTCTG
GCCAACTGTTGATGGAGAGTTTTTGCCTCATAGACCAGAAGAGTTGGTTTACAACCACACTATCGATCCTAACATCAACGTTT
TGTTGGGTACTAACGCTAATGAAGGTGCTTTTATGTTGTTGATGAAGGATATGGTTACTTTCCATCCTTTGAACCCAATTAAT
TTGACTATCCCACACGCTAAGTACATCTTCGGTAAATTGTTCGGTAAAACTTTGATCGATTTCTACTCTGAGAGATATTTGGC
TTCTTTGTCTGCTGATGATTCTGATTCTATTAGATTGGCTGTTACTCAAGCTTTGGGAGATTCTATTTTCACTTGTCCAACTT
ACGCTACTGGTAGAGATTTGATTGCTTCTGGTGTTCCTAACGTTTACGGTTACGTTCAAACTCAAAAGCCAACTCATACTTTG
ATTTCTGTTTCTTCTCAAGCTAAATGGATGTCTAACTTTGCTTCTCATGCTGATACTTTTCCTATGGTTTTCGGTCACCCATT
CAACAAGTTGGATAAGTTCAAAAACGAAGATGTTGTTTTGTCTTTCTTGATGATGGATATCTGGACTAAGTTCGCTAAAGATG
GTAAACCACCTCATATTTCTCACCAAGAGTGGTTGCCTTGGAATTTGTCTGATCCAATTCCTTATCCAACTATGATTTTGGAT
TCTAACAAAATCGGTTTGATCGAAACTAAGTCTGCTGAGTTTTGTGCTAAAAACTGGCCATTCCCTTTGGAGAACTACTATTC
AATGGATTCCGAGAGTGGTGTTTATGCTGAGATCGAGGGTCTAGA

Codon optimized sequence of CCEinc18

GAATTCGAAAACGTCGAGCCAGAGCTTACATTGTCTTCAGGTTCCATCAGAGGTACTTCCGTCGATTTCAGAGGAGTCAAG
GTCTATCAGTTCTTGGGTATTCCATTCGCTGAACCACCTTTGAACGAGTTGAGATTTCAAAAACCAGTTCCTAAGAAACCTTG
GAACGGTGTTTTGTCTGTTAATAAGTGGGGTTCTGCTTGTATGCAACCAGTTTTTCCTGGTTTCAACACTGAATTGCATTTGT
CTGAGGATTGTTTGATCTTGAACGTTTTTACTACTGAAGCTGCTTTCCAAGATAAGCAAAACGGTAAAAAGAACTCTCTTAGA
CCAGTTATGGTTTGGATTCACGGTGGTGGTTTTAACTTCGGTTCTGCTAATACTCCATCTCAATACGATGGTACTCCTATCAC
TGGTTTGAAGGATGTTATCATCGTTTCTATTAACTACAGATTGTCTTCTTTGGGTTTCTTGCATTTGCCAGAGGCTGGTGTTC
CTGGTAACATGGGTTTGTGGGATCAACAATTGGCTTTGAAGTGGGTTAAGGATAACATCGAACACTTCGGTGGTAACCCAAAT
AGAGTTACTATTTTTGGAGAGTCTGCTGGTTCTATGTCTGTTTCTGCTCATATTGTTTCTCCTCACTCTAAGGGTTTGTTTAA
AAACGCTATCATCCAATCTGGTTCTATCTACGATTTGAAAAGATGGGCTCAACCAAAGTTGGCTAAGACTTTGTTGTCTAAGA
TCGGTTGTGAATCTAACGATTACAAGTCTTGTTTGTCTAACTACCAATTCGGTCAATTCTCTGAAGGTTTGACTTTTTGGCCA
ACTGTTGATGGAGAGTTCTTGCCAAACCATCCTGAAGAGTTGGTTTCTAATCACGGTGTTGATCCTAACATTAATGTTTTGTT
GGGTACTGTTGGTAATGAAGGTGCTTTCATGTTGTTGTTGAAGGATTTGGTTACTTTTCATCCATTGAACCCTGTTAATTTGA
CTATTACTCACGCTAAGTACATTATTGGTAAATTGTTCGGTAAAAAGTTGATCGATTTCTACTCTGAGAGATATTTGGCTTCT
TTGCCAGCTGATGATTCTGATGCTATTAGATTGGCTGTTGCTCAAGCTTTGGGAGATACTATTTTGGCTTGTCCTACTTATGC
TTTGGGTAGAGATTTGATTGCTAACGGTGTTCCAAACGTTTACGGTTACGTTCAAACTCAAAAACCTTCTCAAGCTGTTTTGT
TGGTTTCTAACCAGGCTAAGTGGATGGCTAATGTTGCTTCTCATGCTGATGATATTCCAATGGTTTTTGGTCACCCTTTCACT
AAGTTGGATAAGTTTAACAACGAAGATGTTGTTTTGTCTTTCTTGATGATCGATATCTGGACTAAGTTTGCTAGAGATGGTAA
ACCACCTCAAATTTCTCATAGAGAGTGGTTGCCATGGAACTTGTCTGATTCTATTCCATACCCTTCTATGGTTTTGAACTCTA
ATAAGATCGGTTTGATCGAAACTAAGTCTGTTGAGTTTTGTATTAAGAATTGGCCATTCCCTTTGGATAAACCTTACAATATG
GACTTTGAGACAGGTGTTTATGCCAAGATGGAGGAAGATCACGACGAGTTGGGTCTAGA
