# Supplementary material for: High-Resolution Genetic Mapping Combined with Transcriptome Profiling Reveals That Both Target-Site Resistance and Increased Detoxification Confer Resistance to the Pyrethroid Bifenthrin in the Spider Mite Tetranychus urticae
Source: Biology (Basel). 2022 Nov 7;11(11):1630. doi: 10.3390/biology11111630 (PMC9687926; doi:10.3390/biology11111630)
Supplement: Supplementary file 1 [file biology-11-01630-s001.zip › Supplementary figures with captions_proof.pdf]

Supplementary figures

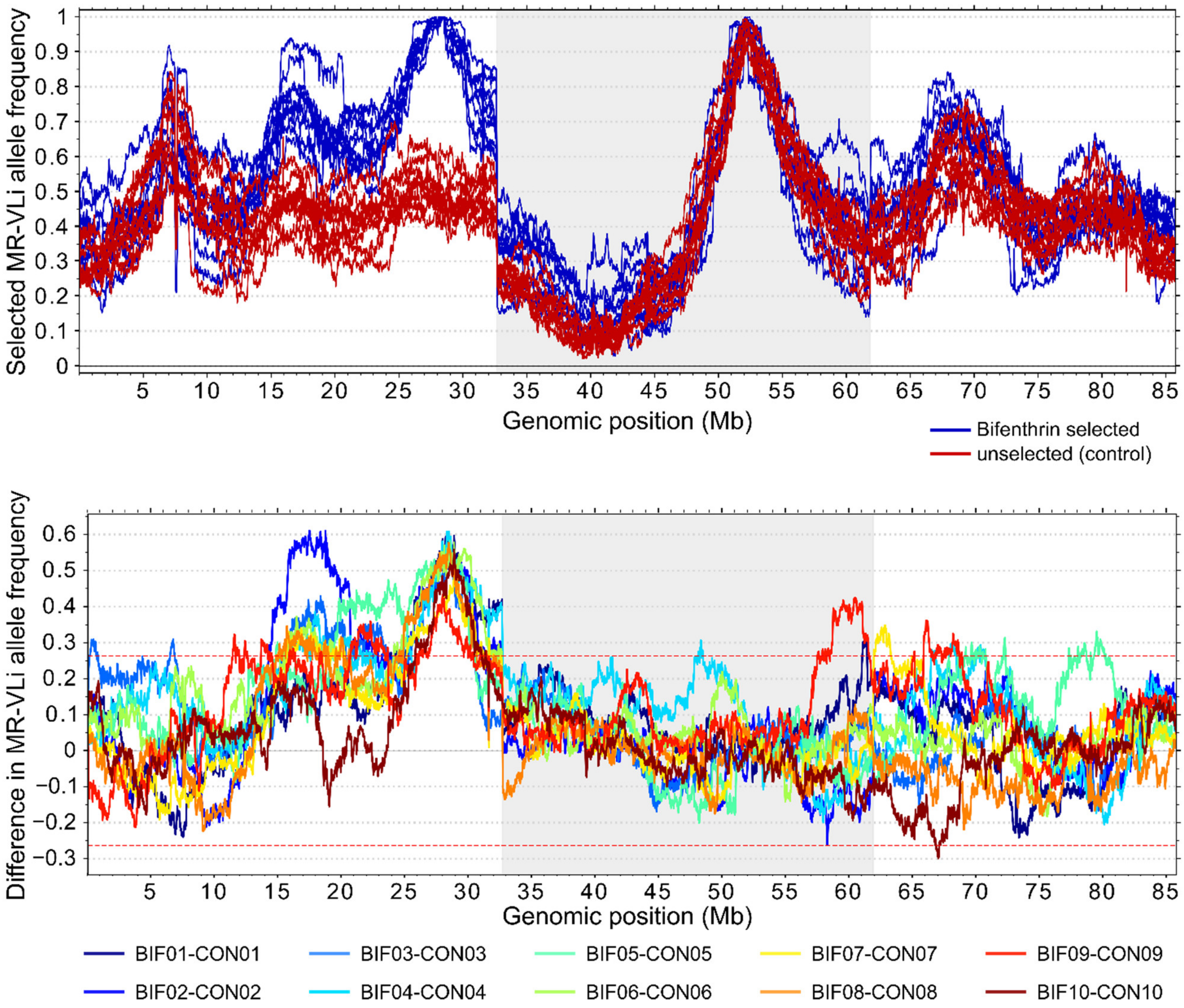

Figure S1. Deviations in allele frequencies of bifenthrin (BIF) selected and unselected (control, CON) populations of the bulked segregant analysis.

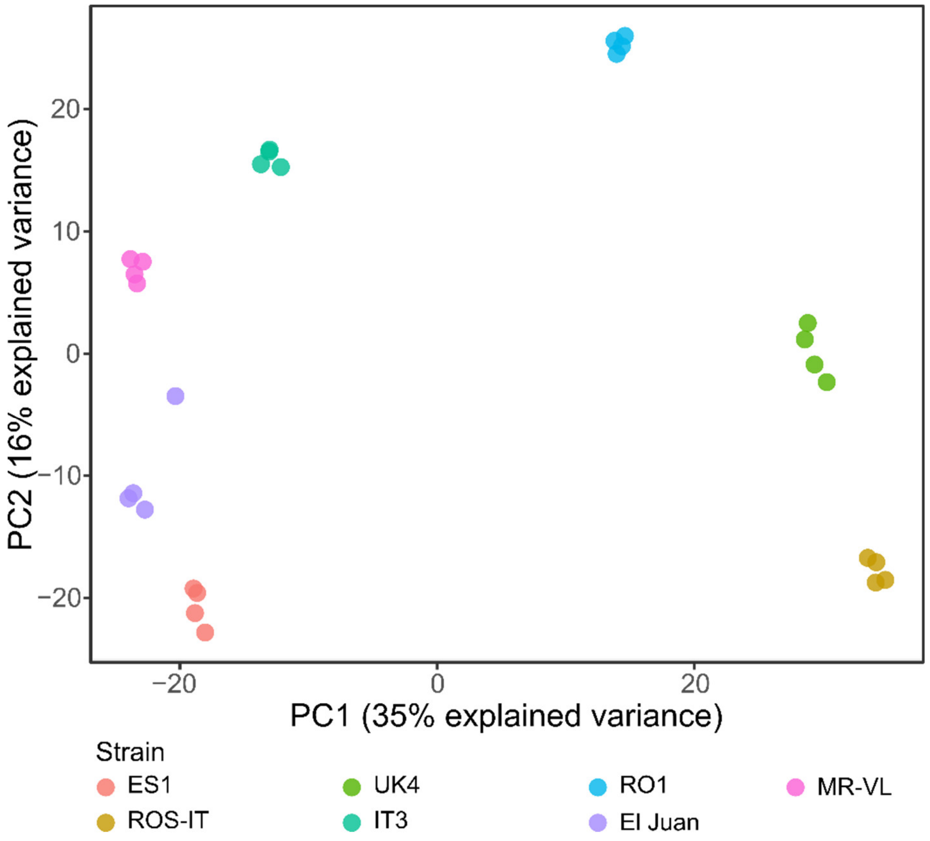

Figure S2. Principal component analysis of the RNAseq expression data of the green strains ROS-IT, UK4 and RO1 and the red strains El Juan, MR-VL, ES1 and IT3. A clear clustering of the replicates per population and separation of the four red strains away from the green strains is observed.

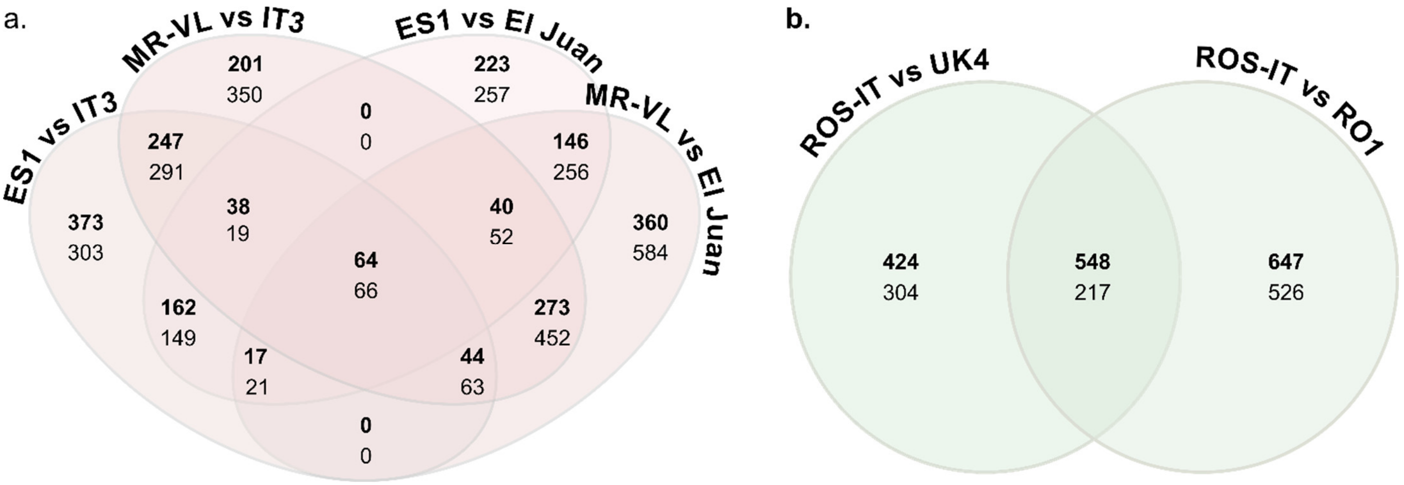

Figure S3. Venn diagrams showing the over-and underexpressed genes with absolute $log_2FC \geq 1$ and p adj < 0.05 in the pairwise comparisons between the susceptible and resistant *T. urticae* strains. The number of overexpressed genes are depicted in bold, the number of underexpressed genes are depicted in normal font. a) the red strain comparisons ES1 vs IT3, MR-VL vs IT3, ES1 vs El Juan and MR-VL vs El Juan. b) the green strain comparisons ROS-IT vs UK4 and ROS-IT vs RO1.

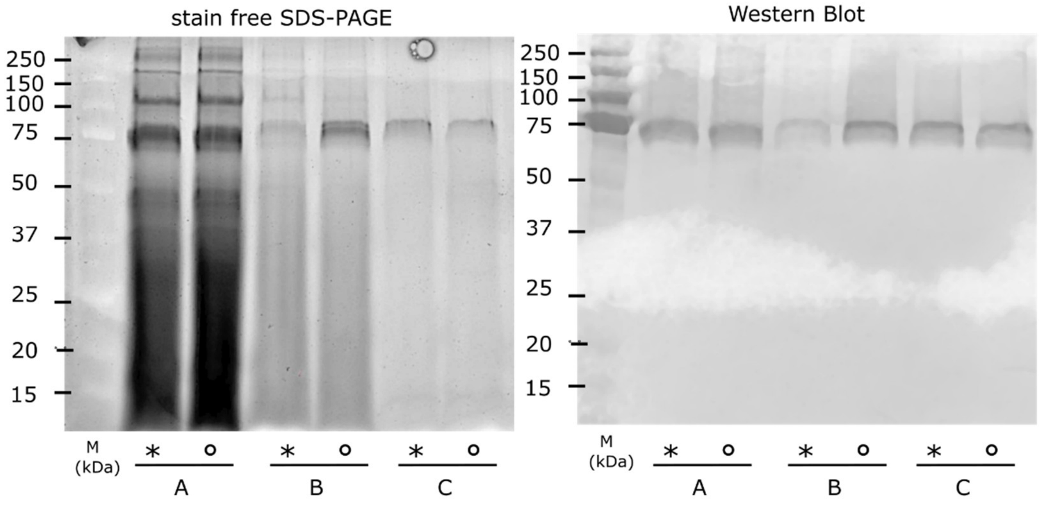

Figure S4. Stain free SDS-PAGE (left panel) and Western blot (right panel) of CCE58 and CCEinc18 along several steps in the expression-purification process. 'A' are samples of concentrated expression medium; 'B' are samples of ammonium sulfate precipitated protein after desalting and buffer exchange; 'C' lanes consist of purified protein. CCE58 samples are indicated with *, CCEinc18 samples are indicated with °. 'M': molecular weight protein marker (Precision Plus Protein Standard).

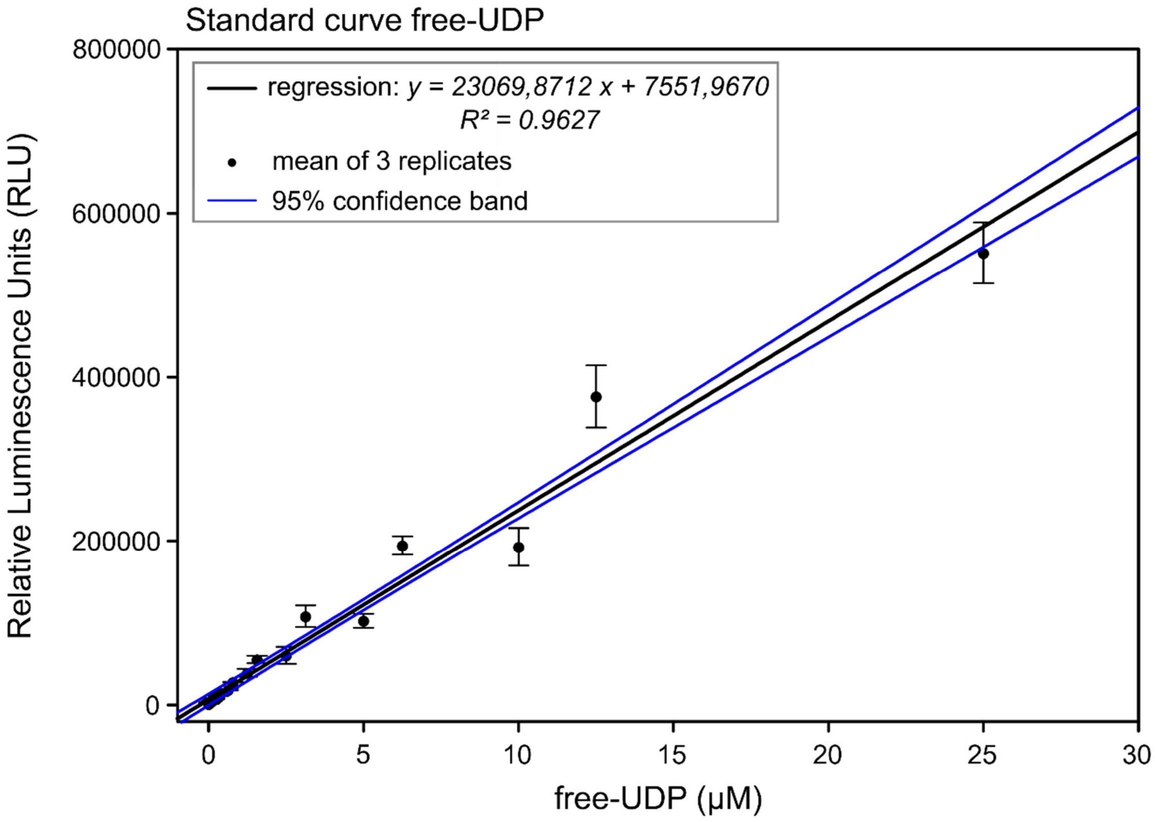

Figure S5. Free-UDP standard curve with Relative Luminescence Units (RLU) in function of free-UDP (range of 0 – 25 µM) measured with a UDP-Glo™ Glycosyltransferase Assay (Promega). Values represent the mean of three replicates ±SD. The plot also shows the regression line ($R^2 = 0.9627$) and the 95% confidence interval.
