# Supplementary figures and images for: High-Resolution Genetic Mapping Combined with Transcriptome Profiling Reveals That Both Target-Site Resistance and Increased Detoxification Confer Resistance to the Pyrethroid Bifenthrin in the Spider Mite Tetranychus urticae
Source: Biology (Basel). 2022 Nov 7;11(11):1630. doi: 10.3390/biology11111630 (PMC9687926; doi:10.3390/biology11111630)

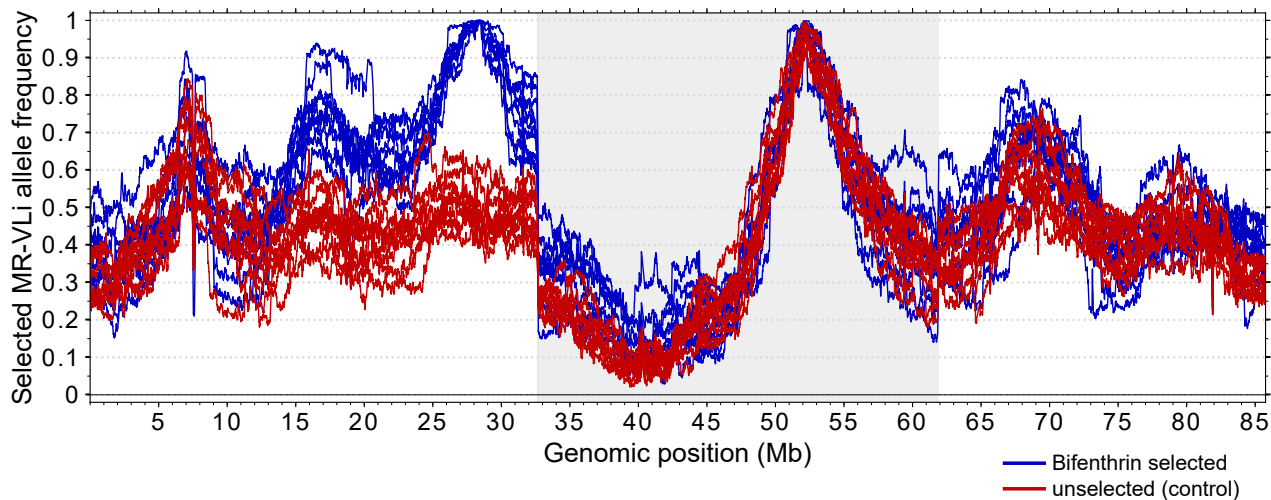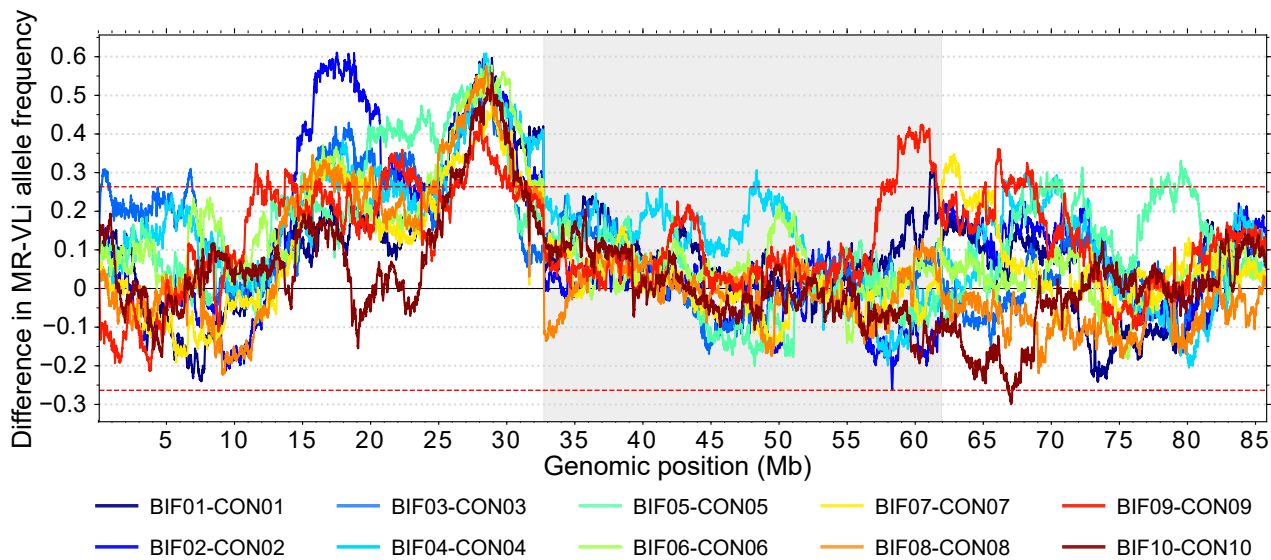

Supplement: Supplementary file 1 [file biology-11-01630-s001.zip › FigS1_Proof.pdf]

PC2 (16% explained variance)

PC1 (35% explained variance)

Strain

ES1

ROS-IT

UK4

IT3

RO1

El Juan

MR-VL

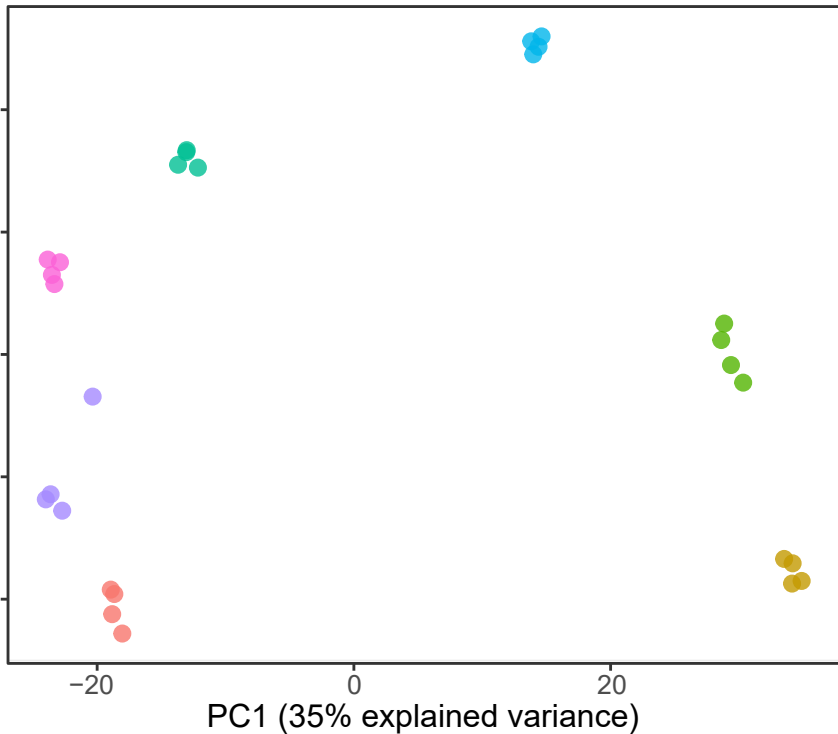

Supplement: Supplementary file 1 [file biology-11-01630-s001.zip › FigS2_Proof.pdf]

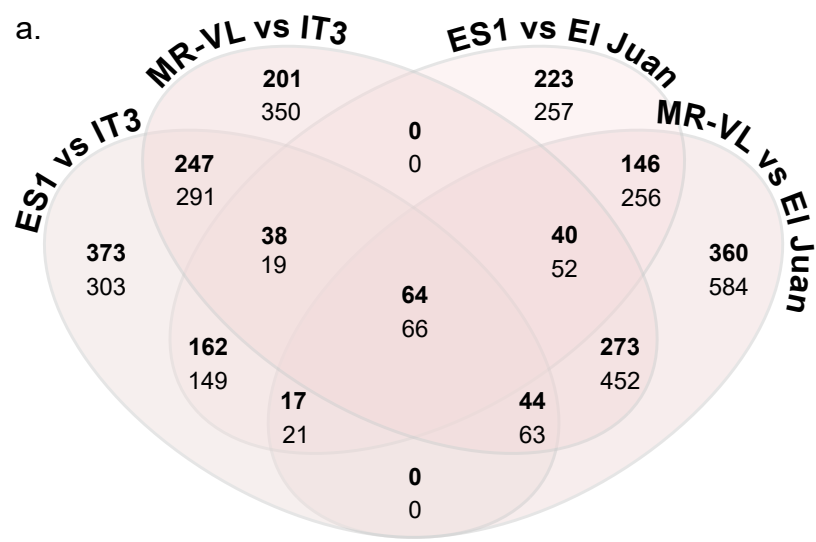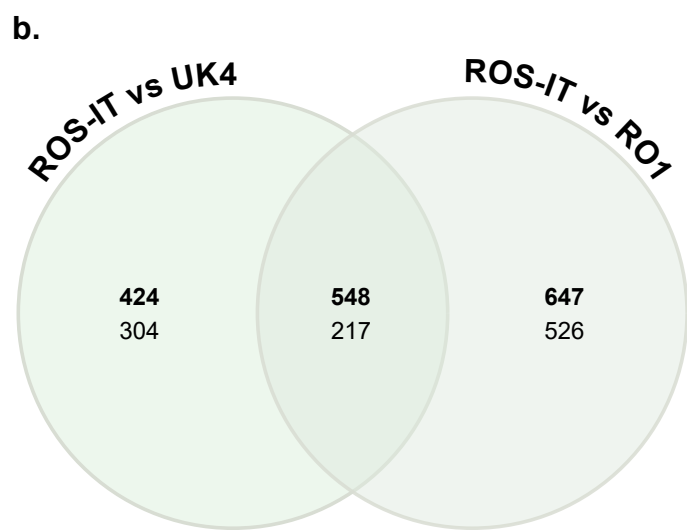

Supplement: Supplementary file 1 [file biology-11-01630-s001.zip › FigS3.pdf]

stain free SDS-PAGE

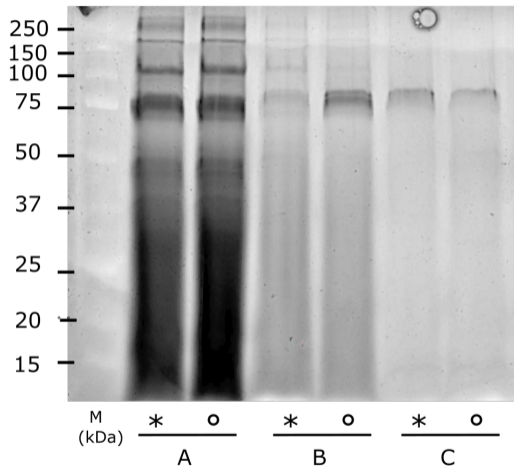

Western Blot

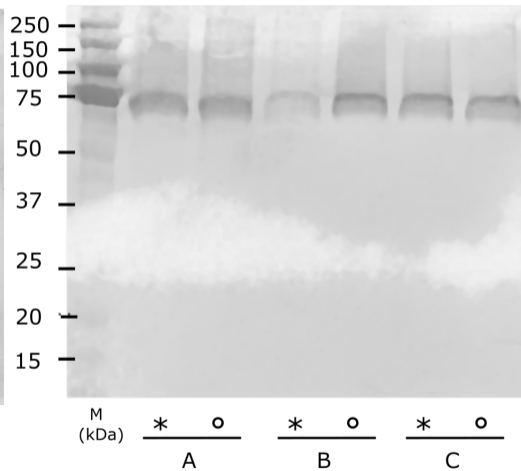

Supplement: Supplementary file 1 [file biology-11-01630-s001.zip › FigS4.pdf]

Standard curve free-UDP

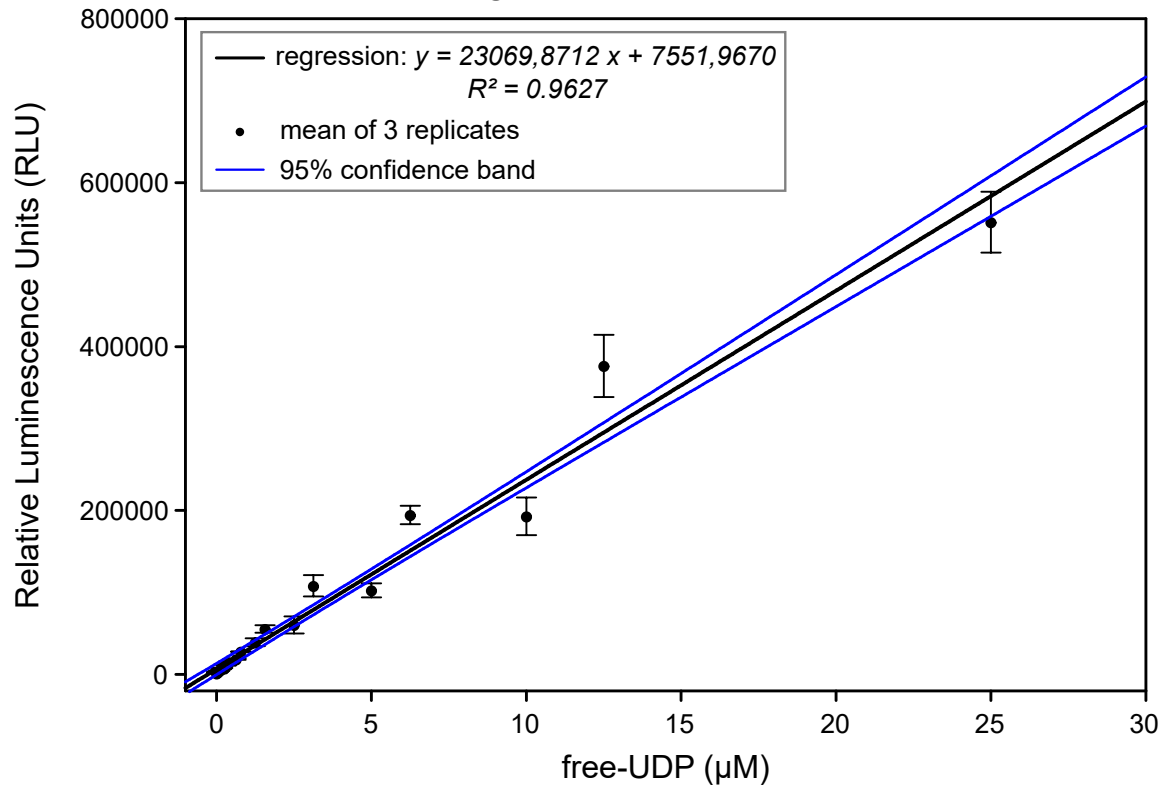

Supplement: Supplementary file 1 [file biology-11-01630-s001.zip › FigS5.pdf]
